# Aqueous Biphasic Dye‐Sensitized Photosynthesis Cells for TEMPO‐Based Oxidation of Glycerol

**DOI:** 10.1002/anie.202200175

**Published:** 2022-03-24

**Authors:** Didjay F. Bruggeman, Annechien A. H. Laporte, Remko J. Detz, Simon Mathew, Joost N. H. Reek

**Affiliations:** ^1^ Homogeneous Supramolecular and Bio-Inspired Catalysis van ‘t Hoff Institute for Molecular Sciences University of Amsterdam Science Park 904 1098 XH Amsterdam The Netherlands; ^2^ Netherlands Organisation for Applied Scientific Research (TNO) Energy Transition Studies Radarweg 60 Amsterdam The Netherlands

**Keywords:** Biphasic Dye Sensitized Photoelectrochemical Cell, Hydrogen, Solar Fuel, Supramolecular Gel, TEMPO-Catalyzed Glycerol Oxidation

## Abstract

This work reports an aqueous dye‐sensitized photoelectrochemical cell (DSPEC) capable of oxidizing glycerol (an archetypical biobased compound) coupled with H_2_ production. We employed a mesoporous TiO_2_ photoanode sensitized with the high potential thienopyrroledione‐based dye **AP11**, encased in an acetonitrile‐based redox‐gel that protects the photoanode from degradation by aqueous electrolytes. The use of the gel creates a biphasic system with an interface at the organic (gel) electrode and aqueous anolyte. Embedded in the acetonitrile gel is 2,2,6,6‐tetramethylpiperidine‐1‐oxyl (**TEMPO**), acting as both a redox‐mediator and a catalyst for oxidative transformations. Upon oxidation of **TEMPO** by the photoexcited dye, the *in situ* generated **TEMPO^+^** shuttles through the gel to the acetonitrile*–*aqueous interface, where it acts as an oxidant for the selective conversion of glycerol to glyceraldehyde. The introduction of the redox‐gel layer affords a 10‐fold increase in the conversion of glycerol compared to the purely aqueous system. Our redox‐gel protected photoanode yielded a stable photocurrent over 48 hours of continuous operation, demonstrating that this DSPEC is compatible with alkaline aqueous reactions.

## Graphical abstract

A biphasic dye‐sensitized photoelectrochemical cell for glycerol oxidation while producing H_2_ is presented. The redox‐gel protects the photoanode from aqueous reaction conditions. The gel layer contained 2,2,6,6‐tetramethylpiperidine‐1‐oxyl and shuttles electrons between the photoexcited dye and glycerol at the biphasic interface. Employment of the redox‐gel showed a 10‐fold increase in glyceraldehyde production compared to the fully aqueous system.

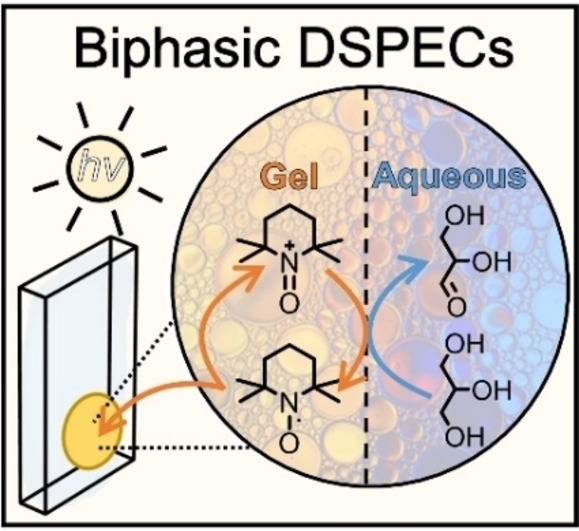

Alternatives to the currently used fossil‐based energy sources are urgently needed to sustain modern‐day human society. Artificial photosynthesis seeks to address this goal with devices that produce chemical fuels using sunlight as energy input. In contrast to photovoltaic electricity, fuels can be stored at large scales to balance solar fluctuations and used for energy‐intensive applications, including transport and metal refining.[^1^, ^2^] Dye‐sensitized photoelectrochemical cells (DSPECs) are an extension of dye‐sensitized solar cells (DSSCs) and produce fuel instead of electricity by introducing catalytic redox reactions at the respective electrodes within the device.^[3]^ DSSCs use molecular dyes, wide‐band gap semiconductors, and redox‐mediators to absorb the light that facilitates a solar‐to‐electrical energy conversion.[^4^, ^5^, ^6^] Redox‐mediators, responsible for dye regeneration in DSSCs, can be substituted for catalysts in DSPECs to realize catalytic chemical conversions.^[7]^ The majority of DSPECs involve water‐splitting and generate hydrogen gas (H_2_)^[8]^ as a potential valuable combustible fuel and chemical feedstock, and oxygen gas (O_2_) as a byproduct. Currently, the efficiencies of such water‐splitting devices are still low (≈1 %).^[9]^ Partly, this is because the oxygen evolution reaction (OER) exhibits a high kinetic barrier,^[10]^ as water oxidation is a four‐electron process. Therefore, there is an interest in exploring alternative oxidative reactions in DSPECs that preferably involve two‐electron processes, *e.g*., alcohol oxidation at photoanodes.[^11^, ^12^, ^13^, ^14^, ^15^, ^16^] Other potential advantages of novel reactions include the wider scope of reaction conditions that become accessible, *e.g*., the solvent and additives used, which have been demonstrated to improve photovoltaic properties in DSSCs.^[17]^ The anodic co‐valorization means that we can couple the production of commercially valuable compounds to H_2_ as fuel, instead of producing O_2_. Although the demand for such fine chemicals (and therefore the coupled H_2_ production) is significantly smaller than that global consumption of fuels, the innate utility of both anodic and cathodic products might facilitate an earlier uptake of this technology. The sustainability of DSPEC‐facilitated photosynthetic production is enhanced further if biomass‐derived feedstocks are valorized by oxidative transformation. In this process, biomass is photochemically reformed to biofuels or fine chemicals through oxidation.^[18]^ A promising candidate for biomass valorization is glycerol, as the oxidized products find application in, for instance, cosmetics and the preparation of polyesters and adhesives.[^19^, ^20^] Current DSPEC designs are incompatible for glycerol conversion as the substrate is water‐soluble and its oxidation requires alkaline (pH≈8.5) conditions.[^21^, ^22^] The prerequisites for glycerol conversion causes stability issues at the photoanode as the ubiquitously employed carboxylic acid—the best performing anchoring group for dyes to metal oxides—is prone to cleavage under aqueous alkaline conditions leading to dye desorption.[^23^, ^24^] More importantly, the photovoltaic properties in aqueous dye‐sensitized systems typically compare poorly with systems using an organic solvent like acetonitrile,^[25]^ incurring losses in power conversion efficiency, lower injection efficiency and shifts of conduction band levels.^[24]^ To make the DSPEC compatible with alkaline aqueous phase conditions required for glycerol oxidation, we envision a biphasic DSPEC system in which the photoanode is protected by an organic phase gel layer. As such, the oxidation reaction takes place in the aqueous phase while an efficient solvent environment for the photoanode (*e.g*, acetonitrile) is maintained.

The biphasic DSPEC (Scheme 1) reported in this communication consists of a mesoporous TiO_2_ photoanode on FTO (fluorine‐doped tin oxide), sensitized with the high oxidation potential thienopyrroledione‐based dye **AP11**.^[26]^ 2,2,6,6‐tetramethyl‐piperidine‐1‐oxyl (**TEMPO**) is a clear choice for the redox‐mediating catalyst because 1) its redox‐mediating ability in quasi‐solid DSSCs using acetonitrile‐ poly (vinylidene fluoride‐*co*‐hexafluoropropylene) (PVDF‐HFP) gel electrolytes,^[27]^ 2) it can act as a redox‐mediating catalyst in acetonitrile‐based DSPECs,[^15^, ^28^] and 3) **TEMPO^+^** has been utilized for the selective aqueous (pH≈8.5) chemical oxidation of glycerol to glyceraldehyde.[^16^, ^22^, ^29^] We created a biphasic system device by applying the acetonitrile‐based PVDF‐HFP gel^[27]^ over the dye‐sensitized photoanode, protecting it from aqueous reaction conditions that lead to device deactivation. Gel‐embedded **TEMPO** can regenerate photooxidized **AP11** at the dye*–*semiconductor interface, generating **TEMPO^+^** that can diffuse through the gel to reach the gel*–*aqueous interface to facilitate glycerol oxidation. As shown by our previous work, it is crucial that *in situ* photogenerated **TEMPO^+^** must migrate away from the semiconductor surface to prevent recombination.^[30]^ Thus, our envisioned biphasic DSPEC design will only function if the **TEMPO** redox‐mediating catalyst resides in the organic‐gel layer. The photogenerated **TEMPO^+^** should diffuse sufficiently fast to the interface to perform the chemical oxidation reaction. Lastly, the gel must not degrade under the employed reaction conditions.

**Scheme 1:**
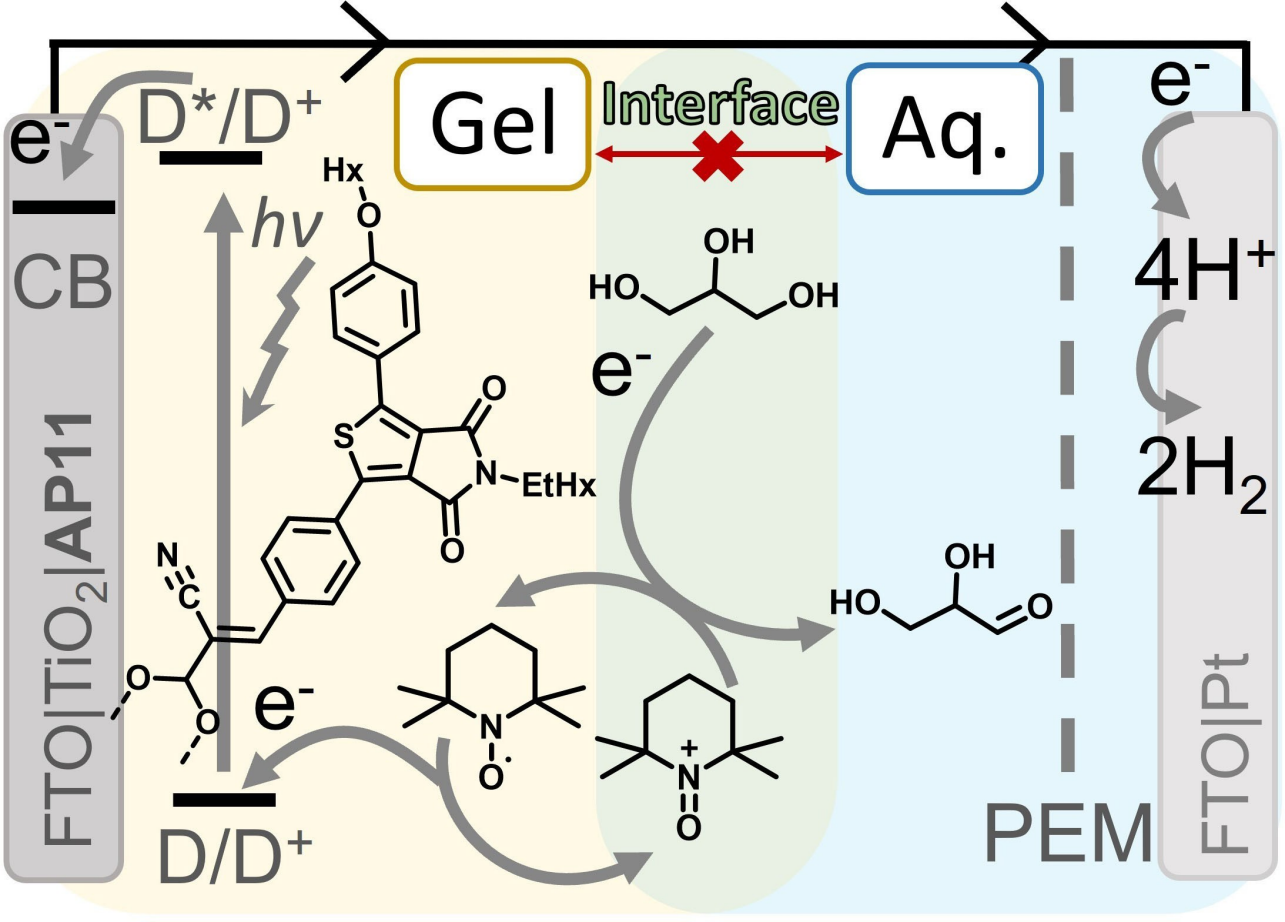
Schematic representation of an aqueous (blue) biphasic DSPEC with a **TEMPO** containing redox‐gel layer (yellow) for glycerol oxidation and simultaneous H_2_ production. Proposed mechanism: 1) irradiation and excitation of a dye, 2) injection of e^−^ in the TiO_2_ CB, 3) e^−^ moves to cathode, 4) **TEMPO** regenerates dye and is oxidized, 5) **TEMPO^+^** oxidizes glycerol to glyceraldehyde at the gel*–*aqueous interface (green) 6) proton reduction to form hydrogen gas as fuel. D=dye D^+^=oxidized dye (1.8 V) D*= excited dye (−0.8 V), e^−^=electrons, CB=conduction band at −0.5 V, **TEMPO**=2,2,6,6‐tetramethylpiperidine 1‐oxyl, **TEMPO^+^** =oxidized **TEMPO**, V *vs*. NHE.

Control experiments were performed to assess the ability of a **TEMPO(BF_4_)** ^[31]^ loaded gel to oxidize glycerol in a biphasic system with glycerol residing in the aqueous layer. The **TEMPO^+^** in this experiment was used to emulate photogenerated **TEMPO^+^** in the envisioned DSPEC. At the same time, the gel stability and the substrate/product distribution over the different layers were probed after 16 hours of reaction time. All acquired experimental samples containing substrates and/or products in respective aqueous/organic solutions were TMS‐derivatized prior to analysis by GC.[^32^, ^33^] We found that the **TEMPO^0/+^** strongly preferred (Table 1, Entry 1, 97 %) to remain in the gel phase. This behavior was independent of the gel layer thicknesses (Table S3, S4). The glycerol substrate and glyceraldehyde product (Table 1, Entries 2 and 3) demonstrated complementary solubility, with 85 % and 87 % residing in the aqueous phase, respectively. The orthogonal solubility of components in this system—redox‐mediator in acetonitrile, substrate/product in aqueous—also simplifies product retrieval. Importantly, while these components are mainly in separate phases, efficient selective chemical oxidation of glycerol in the gel*–*aqueous system was confirmed, with only a ≈50 % decrease in conversion (16.2 %) compared to a fully homogeneous mixed reaction mixture (29.8 %). We found that the gel stayed intact, and the system remained biphasic over several days, easily accommodating the 23 to 48‐hour time‐course of DSPEC experiments (see below). As such, the PVDF‐HFP gel system displays the desired properties to construct the biphasic DSPEC (for more details, see Supporting Information, Table S2–S5).[^32^, ^33^] Notably, substrates that show a preference for organic layers are proven to be not suitable for such a system (Table S7 and S8).

**Table 1:** Overview of the relative distribution over a 1 : 1 biphasic system of glycerol (1 eq.) and **TEMPO(BF_4_)** (1.5 eq.) and conversion of glycerol to glyceraldehyde by **TEMPO(BF_4_)** at *t*=16 hours. Biphasic systems consist of **TEMPO(BF_4_)** in the acetonitrile‐based gel and glycerol in the aqueous layer. Conversion and distribution over various gel thickness are shown in Table S3. Data is analyzed with GC after TMS‐derivatization.

Entry	Compound	Gel phase [%]	Aqueous phase [%]
1	**TEMPO^+/0^**	97	3
2	Glycerol	15	85
3	Glyceraldehyde	13	87

Preliminary experiments revealed that a minimum redox‐gel thickness of 3 mm over the photoanode is needed in the DSPECs to obtain an even, reproducible and stable gel layer coverage over the entire surface of the photoanode. Therefore, the impact of gel layer thickness on the photovoltaic properties was assessed in sandwich‐cell quasi solid‐state DSSCs using redox‐gels to confirm sufficient (re)generation of the photooxidized dye and **TEMPO^+^**. These quasi solid‐state DSSCs were compared to a typical DSSC based on acetonitrile‐based **TEMPO^0/+^** electrolyte. The construction of the sandwich DSSC is detailed in the Supporting Information and Figure S5, and the photovoltaic parameters under irradiation with the 100 mW cm^−2^ LED white light source (Figure S6) are presented in Table 2. A typical **AP11**‐sensitized DSSCs (Table 2, Entry 1) featuring an acetonitrile liquid electrolyte exhibits a short‐circuit photocurrent (*J*_sc_) of 3.8 mA cm^−2^ and an open‐circuit voltage (*V*_oc_) of 0.62 V. Electrolyte substitution with the **TEMPO**‐gel (Table 2, Entry 2) yielded a ≈10 % reduction in *J*_sc_ and minimal impact to *V*_oc_. Increasing the gel thickness from 60 μm to 1, 2 or 3 mm resulted in an approximately similar reduction of photovoltaic performance (Table 2, Entries 3–5). The DSSC with the redox‐gel thickness of 3 mm experienced a reduction in *J*_sc_ to 2.6 mA cm^−2^ (from 3.8 mA cm^−2^) while the *V*_oc_ reduced to 0.41 V (from 0.61 V). The lower performance is a result of increased distance between the electrodes leading to diffusion limitations and, as a result, increased recombination at the semiconductor*–*gel interface due to the accumulation of oxidized species at the photoanode, in line with what was previously reported.[^34^, ^38^] Furthermore, the gel increases cell resistance due to low ion diffusion that leads to a lower Fill Factor (*FF*). The combined effects contribute to a decrease in *η* for the 3 mm gel DSSC compared to the liquid 0.060 mm analog (by 70 %).^[35]^ However, it still produces sufficient *J*_sc_ to generate **TEMPO^+^** for glycerol oxidation in DSPECs. Importantly, future optimization may focus on the generation of devices with smaller gel layers.

**Table 2:** Photovoltaic parameters of the *n*‐type quasi solid‐state FTO|TiO_2_|**AP11** (active area 0.19 cm^2^) sandwich DSSCs with a standard deviation of *N*=3 in brackets. CE= FTO Pt electrodeposited, electrolyte: 1.2 M LiTFSI, 1.0 M **TEMPO** and 0.1 M **TEMPO(BF_4_)** with 10 % wt PVDF‐HFP in acetonitrile. Data was obtained from *J–V* measurements performed under a 100 mW cm^−2^ LED white light source with a 0.07 cm^2^ mask. a) Surlyn or b) Teflon spacer.

Entry	Redox‐gel [mm]	*V*_oc_ [V]	*J*_sc_ [mA cm^−2^]	*FF*	*η* [%]
1^a^	0.060 (liquid)	0.62 (±0.003)	3.8 (±0.11)	0.68 (±0.01)	1.59 (±0.07)
2^a^	0.060	0.61 (±0.007)	3.4 (±0.14)	0.60 (±0.08)	1.24 (±0.02)
3^b^	1.0	0.46 (±0.019)	2.8 (±0.36)	0.44 (±0.01)	0.57 (±0.06)
4^b^	2.0	0.43 (±0.020)	2.6 (±0.41)	0.42 (±0.05)	0.46 (±0.05)
5 ^b^	3.0	0.41 (±0.059)	2.6 (±0.32)	0.45 (±0.02)	0.49 (±0.02)

Next, we explored the light‐driven oxidation of glycerol to glyceraldehyde in a DSPEC. DSPECs were assembled with enlarged WEs (FTO|TiO_2_|**AP11**, masked size 0.64 cm^2^) and CEs (FTO|Pt), which were prepared similar to the (photo‐)electrodes used in DSSCs (see above). The electrodes were placed in a DSPEC photoreactor (Figure 1A) composed of two Teflon compartments (photoanodic for glycerol oxidation and cathodic for H_2_ production) separated by a Nafion‐117 membrane. The redox‐gel containing the **TEMPO** (1.0 M) was (hot) drop‐casted on the photoanode yielding a protective gel layer with a thickness of 3 mm. After the gelation was complete (≈0.5 hours), the anolyte containing 0.1 M glycerol was introduced (sat. NaCl, NaHCO_3_ pH 8.3, 3 mL; see Figure 1B). Chronoamperometric experiments were performed by illuminating the photoanode with a 100 mW cm^−2^ white LED (Figure S6) for 23 hours, using a small bias potential of 0.1 V *vs*. Ag/AgCl (Figure S7A). Aliquots of the anodic reaction mixture were TMS derivatized and analyzed by GC to probe the conversion, and the results are shown in Figure 2B and summarized in Table 3. Control experiments were performed using the redox‐gel DSPEC without illumination, using a redox‐gel DSPEC without **TEMPO** as a redox‐mediating catalyst, and by using the DSPEC without gel.

**Figure 1:**
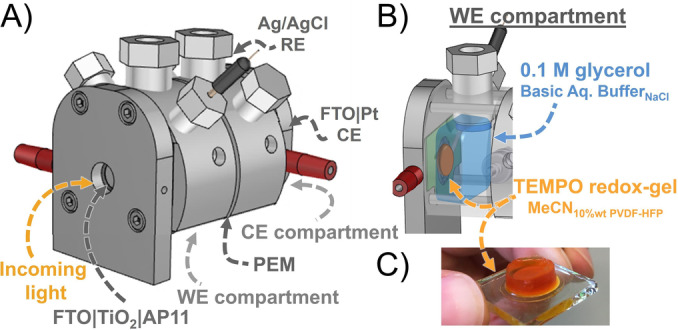
A) Overview of the photoreactor, B) WE compartment of photoreactor containing the photoanode FTO|TiO_2_|**AP11** overlayed with 1.0 M **TEMPO** 3 mm redox‐gel (10 % wt PVDF‐HFP, 1.2 M LiTFSI in acetonitrile), and the bulk aqueous glycerol solution, C) Image of the **TEMPO** redox‐gel.

**Figure 2:**
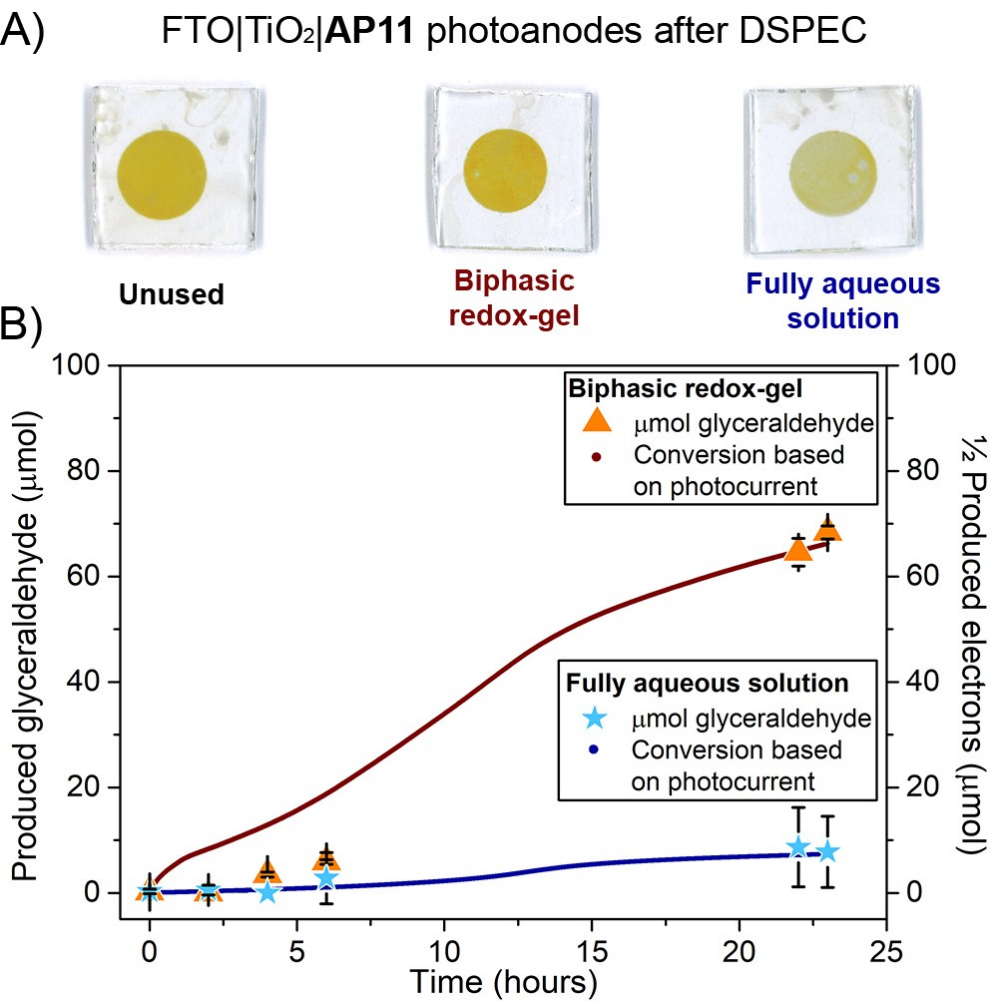
A) Images of used photoanodes in 23 hours DSPEC experiments, and B) GC quantification of light‐driven glyceraldehyde production in a DSPEC with 3 mm **TEMPO** redox‐gel layer (orange) and a full aqueous **TEMPO**‐based system (blue) with the anticipated amount of glyceraldehyde based on photocurrent (redox‐gel: brown, aqueous dark blue). The integration of half the photocurrent determines the number of electrons to account for two electrons needed per oxidation reaction. A bias potential of 0.1 *vs*. Ag/AgCl was applied on the WE and the system was illuminated with a 100 mW cm^−2^ white LED light source (masked size 0.64 cm^2^). Experimental details are found the Supporting Information and Table 3.

**Table 3:** Light‐driven glycerol oxidation reactions using redox‐gel biphasic DSPEC performed for *t*=23 hours and control experiments. The biphasic gel DSPEC: WE electrode compartment consisted of a FTO|TiO_2_|**AP11** WE (active area 0.74 cm^2^) with a 1.0 M **TEMPO** 3 mm redox‐gel layer (10 % wt PVDF‐HFP, 1.2 M LiTFSI in acetonitrile) and filled with 0.1 M glycerol aqueous solution (sat. NaCl, NaHCO_3_ pH 8.3, 3 mL). An Ag/AgCl RE which was placed close to the WE. The CE compartment was separated by a Nafion‐117 membrane and consisted of a FTO|Pt CE and was filled with 1.0 M AcOH in acetonitrile (3 mL). A bias potential on the WE of 0.1 *vs*. Ag/AgCl was employed and the system was illuminated with a 100 mW cm^−2^ white LED light source (masked size 0.64 cm^2^). Differences in control experiments are indicated. Product analysis was measured by GC after TMS‐derivatization. * Faradaic efficiency was not obtained for system with photocurrent near 0 μmol. Images of photoanodes are shown in Table S10.

Entry	Conditions	Glyceraldehyde produced [μmol]	Conversion based on photocurrent [μmol]	Faradaic efficiency [%]
1	Biphasic gel	68.3	66.2	≈100
2	w/o light	0	<0	–*
3	w/o **TEMPO**	0	1.00	–*
4	w/o gel (aqueous)	7.60	7.40	≈100

The experiment where the biphasic redox‐gel DSPEC system was irradiated for 23 hours (Table 3, Entry 1) yielded 68.3 μmol of glyceraldehyde. This translates to a 100 % Faradaic efficiency based on the photocurrent produced. Negligible photocurrent is produced in control experiments that exclude light or **TEMPO** (Figure S7B), and no glyceraldehyde was formed in these experiments as judged by GC analysis. As expected, both light and **TEMPO** are necessary to create an operational cell capable of the glycerol oxidation reaction (Table 3, Entries 2 and 3). The redox‐gel DSPEC is compared to a fully aqueous DSPEC (Table 3, Entry 4 and Figure 2B), which contains a saturated solution of **TEMPO** (≈0.5 M). Although the **TEMPO** concentration is lower in the fully aqueous DSPEC, our previous studies have shown that DSPECs with **TEMPO** concentrations as low as 0.01 M in acetonitrile still work well and outperform the DSPEC based on the aqueous anolyte.^[30]^ Furthermore, it is known that wettability issues for **TEMPO**‐based aqueous electrolytes leading to poor pore infiltration are not an issue.^[36]^ While both DSPECs—the **TEMPO** redox‐gel and the completely aqueous redox system—demonstrate a Faradaic efficiency for glyceraldehyde production of ≈100 %, the photocurrent produced by the **TEMPO** redox‐gel based DSPEC is ≈10‐fold higher than that of the aqueous system. In line with this, the improved photocurrent results in a 10‐fold increase in glyceraldehyde production.

The DSPEC photoanodes and redox‐gels were investigated after the experiments to gain insight into the stability of the device. The redox‐gel preserved its bright orange color after 48 hours of chronoamperometric illumination (Figure 1C), consistent with the control experiments described in Table 1 that reveal only a small fraction (6 %) of all **TEMPO** was found in the aqueous layer samples (Figure S8). The water present in the gel layer only increased by 1 % (Table S6) after a 48‐hour experiment. Quantitative and qualitative colorimetric analysis of FTO|TiO_2_|**AP11** photoanodes (Figure 2A and Table S10) is performed, as the disappearance of the yellow color indicates detachment or decomposition of the **AP11** dye due to the lingering of the dye in the oxidized state.^[31]^ DSPEC photoanodes featuring gel layers without the **TEMPO** redox‐mediator were completely bleached (Table S10, Entry 3). As expected, slight desensitization of the photoanodes used in experiments of the aqueous system was noted, in line with cleavage of the carboxylic acid linker of the **AP11** dye under alkaline aqueous conditions.^[23]^ Soaking studies were performed in the dark to avoid dye decomposition by over‐illumination, to study the impact of alkaline aqueous conditions, and confirmed the leaching of **AP11** of TiO_2_ in such an environment (Table S9). Interestingly, the yellow color was retained in the photoanode protected with the redox‐gel, indicating that the gel prevents cleavage of the dye, and there is sufficient redox‐mediator in the gel system present to avoid the photodecomposition of the dye. Furthermore, the redox‐gel based DSPEC still performs after 24 hours, whereas the fully aqueous‐based DSPEC no longer generates photocurrent after ≈15 hours (Figure S7B).

Interestingly, the system needs several hours before the reaction proceeds with 100 % Faradaic efficiency. We surmise that the DSPEC builds up the **TEMPO^+^** concentration in the gel based on the diffusion through the redox‐gel layer to a steady‐state required to allow oxidation reactions to occur at the gel‐electrolyte interface. Therefore, we performed 48‐hour experiments to confirm the steady‐state in **TEMPO^0/+^** and further probe the system‘s stability. These experiments show (Figure S11–S13) that the DSPEC with a gel layer tends to stabilize after approximately 20 hours of irradiation, after which a stable photocurrent density of ≈0.18 mA cm^−2^ is observed, producing glyceraldehyde with a near 100 % Faradaic efficiency. In a separate experiment, the redox‐gel based DSPEC was illuminated for 7 hours to quantify the amount of H_2_ formed during the experiment, and this experiment shows (Figures S9 and S10) a high Faradaic efficiency in H_2_ formation (of ≈90 %). This experiment ultimately shows that the gel‐protected biphasic DSPEC allows simultaneous fuel formation and glycerol oxidation.

In conclusion, we have demonstrated the selective light‐driven **TEMPO**‐mediated oxidation of glycerol to glyceraldehyde coupled to the production of H_2_ in a biphasic‐DSPEC utilizing a gel‐protected photoanode. The **TEMPO** redox‐mediating catalyst is photooxidized to **TEMPO^+^** in the acetonitrile‐based gel and oxidizes glycerol at the gel*–*aqueous interface. The presence of the gel layer on the photoanode increases the photocurrent density and product formation by 10‐fold compared to the aqueous DSPEC system. In the absence of the gel layer, the photoanode decolorizes due to dye detachment under these conditions. The improved efficiency and high stability of the redox‐gel‐based DSPEC opens up the possibility for the photoconversion of other solely water‐soluble including biomass‐derived substrates such as cellulose, which also require relatively harsh conditions.^[37]^ Furthermore, product collection from the aqueous solution is improved thanks to the embedding of the active redox‐mediating catalyst in the redox‐gel. Most excitingly, the concept of employing a redox‐gel to enhance the stability of aqueous DSPECs can be considered in the context of water‐splitting DSPECs. On top of that, we are currently investigating if the redox‐gel can prevent recombination in such cells by physically separating the water oxidation complex from the photoanode surface.

## Conflict of interest

The authors declare no conflict of interest.

## Supporting information

As a service to our authors and readers, this journal provides supporting information supplied by the authors. Such materials are peer reviewed and may be re‐organized for online delivery, but are not copy‐edited or typeset. Technical support issues arising from supporting information (other than missing files) should be addressed to the authors.

Supporting Information

## Data Availability

The data that support the findings of this study are available from the corresponding author upon reasonable request.
