# Supplementary material for: Aqueous Biphasic Dye‐Sensitized Photosynthesis Cells for TEMPO‐Based Oxidation of Glycerol
Source: Angew Chem Int Ed Engl. 2022 Mar 24;61(21):e202200175. doi: 10.1002/anie.202200175 (PMC9401026; doi:10.1002/anie.202200175)
Supplement: Supplementary file 1 — Supporting Information [file ANIE-61-0-s001.pdf]

Angewandte Chemie
Eine Zeitschrift der Gesellschaft Deutscher Chemiker

Supporting Information

Aqueous Biphasic Dye-Sensitized Photosynthesis Cells for TEMPO-Based Oxidation of Glycerol

*D. F. Bruggeman, A. A. H. Laporte, R. J. Detz, S. Mathew, J. N. H. Reek**

Table of Contents

Experimental Procedures 3

Materials and devices 3

Preparation of redox gel based on acetonitrile containing PVDF–HFP polymer. 3

Procedure for gas chromatography samples using a TMS-derivatization method. 3

Standard reaction setup chemical oxidation reactions using **TEMPO(BF$_4$)** as a chemical oxidant 4

A standard setup for distribution experiments of **TEMPO(BF$_4$)** and glycerol in a biphasic system 4

Cleaning of the FTO electrodes 4

Preparation of the photoanode FTO|TiO_2 4

Preparation of the platinum counter electrodes 5

Construction of dye-sensitized solar cells (DSSCs) 5

Illumination setup for of the dye-sensitized photoelectrochemical cells (DSPECs) 5

Quantification of water in redox-gel. 6

TEMPO$^{0/+}$, glycerol, and glyceraldehyde exchange and product conversion between (organic)gel layer and aqueous layer. 6

Figures and Tables

Table S1: Determined gas chromatography retention times for all used compounds 7

Figure S1. Gas chromatography calibration curve for glycerol 7

Figure S2. Gas chromatography calibration curve for glyceraldehyde 7

Figure S3. Gas chromatography calibration curve for **TEMPO** 7

Figure S4. Gas chromatography calibration curve for H_2 in 2 mL air 8

Table S2. Images of setup for chemical oxidation reactions with **TEMPO(BF$_4$)** in gel biphasic 8

Table S3. Overview chemical oxidation reactions of **TEMPO(BF$_4$)** and glycerol in gel biphasic systems 9

Table S4. Overview of biphasic exchange studies of **TEMPO(BF$_4$)** in gel biphasic systems: 10

Table S5. Overview of biphasic exchange studies glycerol in gel biphasic systems 12

Figure S5. Schematic overview of the assembly of a DSSC with Teflon spacer 13

Figure S6. Reference spectrum of used illumination source 14

Figure S7A. Initial chopped light experiments of the various DSPEC systems. 14

Figure S7B. Overview of the photogenerated current density in all DSPEC measurements during 24 hours. 14

Figure S8. Graph of **TEMPO** present in the aqueous layer in DSPEC experiments obtained over 24 hours 15

Table S6. Increase in water content of gel during 48 hours DSPEC measurements 15

Figure S9A. H_2 measurement in DSPEC experiments over a period of 7 hours. 16

Figure S9B. H_2 and 1/2 electrons produced in DSPEC experiments over a period of 7 hours. 16

Figure S10. Overview of the photogenerated current density in H_2 DSPEC measurements during 7 hours. 16

Figure S11A. GC Chromatogram for the sampling of long-term DSPEC experiments measured over 48 hours. 17

Figure S11B) GC quantification of substrate to product formation corresponding to Figure 11A 17

Figure S12A. Overview of the photogenerated current density produced in DSPECs during 48 hours 18

Figure S12B. Graph of glyceraldehyde and 1/2 electrons produced in DSPECs during 48 hours. 18

Figure S13A. **TEMPO** in the aqueous layer in two long-term DSPEC experiments measured over 48 hours. 18

Figure S13B. Glycerol and glyceraldehyde in the aqueous layer DSPEC experiments measured over 48 hours 18

Table S7. Overview of biphasic exchange studies and oxidation studies with **TEMPO(BF$_4$)** of benzyl alcohol 19

Table S8. Overview of biphasic exchange studies and oxidation studies with **TEMPO(BF$_4$)** of HMF 19

Table S9. Dye leaching experiment with ø 0.5 cm FTO|TiO_2|**AP11** in alkaline or organic anolytes. 19

Table S10. Images of photoanodes used in DSPEC experiments 20

References 20

Author Contributions 20

Experimental Procedures

Materials and devices

All reagents and solvents were obtained from Sigma-Aldrich, Fluorochem, or VWR and used without purification unless described otherwise. Reactions were performed under atmospheric conditions unless otherwise noted. **AP11**[1] and **TEMPO(BF$_4$)**[2] were used from previous studies and synthesized according to literature procedures. Dye-sensitized solar cells (DSSCs) and dye-sensitized photoelectrochemical cells (DSPECs) were measured on a P211 potentiostat (Zahner) and illuminated with a LED light source (Zahner, TLS3, 100 mW cm^{-2}), with the spectral output given in Figure S6. Gas chromatography (GC) of liquid samples were performed on a Trace GC Ultra machine (Interscience) with an RTX-1 column (30 m, 0.25 mm internal diameter, 0.25 µm film thickness), inlet temperature 70°C hold 2.00 minutes ramp 10°C min^{-1} to 340°C with 1-hexanol added as an external standard due to the uneven distribution over the biphasic system. (Table S1, Calibration curve, Figure S2A, B, and S3). Hydrogen gas qualification was measured on a Gas Chromatograph (Shimadzu Nexis GC-2030) with a 5Å mol sieve column (60 m, 0.32 mm internal diameter, 25 µm film thickness) with an inlet temperature of 40°C and a flow argon rate of 8.0 mL min^{-1} (Calibration curve, Figure S4). Water was quantified with an 831 KF Coulometer (Metrohm) with Hydranal solution.

Preparation of redox gel based on acetonitrile containing PVDF–HFP polymer.

The acetonitrile-based PVDF–HFP polymer gel was prepared by literature procedures.[3] Poly(vinylidene fluoride-*co*-hexafluoropropylene) (PVDF–HFP, M_W=400k, M_n=130k) (product 427160, Sigma–Aldrich, 10%wt, 0.393 g), lithium bis(trifluoromethanesulfonyl)imide (LiTFSI) (1.2 M, 1.44 g) were added to acetonitrile (5 mL) The mixture is heated to 80°C overnight yielding a viscous clear liquid and kept at 80°C until used. **TEMPO** and/or **TEMPO(BF$_4$)** is added fresh to the hot mixture before every experiment and kept at 80°C until dissolved. The redox-gel was formed by letting the mixture cool down to room temperature.

Procedure for gas chromatography samples using a TMS-derivatization method.

All GC samples (calibration curves/determination of retention times of 1-hexanol, glycerol, glyceraldehyde, dihydroxyacetone, **TEMPO** (*N*-oxyl/ oxoammonium exhibit identical retention times, confirmed with GC–MS), biphasic measurements, and DSPEC measurements) were prepared in the same manner, utilizing BSTFA as a trimethylsilyl (TMS)-based derivatization procedure for hydroxy groups.[4] TMS-derivatization of hydroxy groups generally increases the volatility of alcohol and carbonyl compounds, making them suitable for GC analysis. Although this is unnecessary for **TEMPO**-based compounds, we treated all samples similarly to generate consistency. Acetonitrile (280 µL) was added to a GC-vial, after which 1-hexanol (20 µL, 100 mM in acetonitrile) was added as an external standard. Subsequently, the acquired experimental sample containing the substrate and/or product in aqueous/organic solution (20 µL) is added. N, O-bistrifluoroacetamide (BSTFA) (300 µL) is added to the mixture, and trifluoroacetic acid (TFA) (2 µL) is added last. The left-open vial is heated to 50°C for 30 min. Subsequently, BSTFA (400 µL) was added to the vial, and the sealed vial was heated to 80°C for 30 min. The vial was cooled to room temperature, after which 1 µL of the derivatized solution was injected into GC. Peak identification and assignment were performed using a combination of published data[5–8] and by analyzing pure, derivatized, commercially-available samples, see Table S1 for retention times. Previous research[5–8] established that glycerol, glyceraldehyde, and dihydroxyacetone can form a multitude of structures leading to various peaks in the chromatogram for incomplete TMS-derivatization, acetals, monomers, and dimers (through hemiacetal and hemiketal bond formation) depending on the amount and type of derivatizing agent and water present. 1-hexanol, glycerol, and dihydroxyacetone each exhibit a single peak corresponding to the TMS-derived compound, confirmed with GC–MS. However, the quantification of the pure glyceraldehyde gave two peaks with retention times 11.3 and 11.7 (min). The two peaks were analyzed with GC–MS, after which we surmised that these peaks correspond to the TMS-protected keto- (Glyceraldehyde_A) and enol- (Glyceraldehyde_B) tautomers of glyceraldehyde. A calibration approach involving the sum of the peak areas was used.[8] However, other possibilities such as incomplete TMS-derivatization, acetals, monomers, and dimers (through hemiacetal and hemiketal bond formation) of the TMS derivatives glyceraldehyde may also be suitable since the exact species is often hard to determine, as discussed by A. Parodi, E. Diguilio, S. Renzini, I. Magario in *Carbohydrate Research* **2020**, *487*, 107885. Since the qualification on the TMS-glyceraldehyde species formed during the aqueous derivatization process is generally very strenuous, we would like to refer the reader to the previous mentions reference for more information and in-depth discussion on the GC analysis of these compounds.[8]

Standard reaction setup chemical oxidation reactions using TEMPO(BF$_4$) as a chemical oxidant

The standard setup for the biphasic chemical oxidation experiments was a two-layered system of gel/aqueous solution ratios of 1:3, 2:3, and 3:3 (in v/v 100 µL) (images of experimental setup Table S2, Entry 3–5). A fully mixed system (400 µL of 0.15 M **TEMPO(BF$_4$)**, 0.1 M glycerol, Table S2, Entry 1) was used as a control. The ratios oxidant and substrate were kept similar, yielding a theoretical maximum conversion of ~80% (Table S3) since the oxidation from glycerol to glyceraldehyde is a two-electron process while **TEMPO(BF$_4$)** is a one-electron oxidant. As a control, bilayer systems lacking PVDF–HFP were made to analyze the organic layer. The redox-gel contained **TEMPO(BF$_4$)** (0.15 M) and was obtained as described before. The gel (100 µL, 200 µL, and 300 µL) was added to a vial after which 300 µL glycerol (0.03 M, 0.05 M or 0.1 M in aqueous solution sat. NaCl, NaHCO3 pH 8.3) was slowly added on top of the redox-gel. The equivalent oxidant to the substrate was kept 1.5:1. After 16 hours, aliquots of the aqueous layer and/or the organic layer were obtained and derivatized as described above, prior to GC analysis to study the effect of gel thickness on glycerol oxidation.

A standard setup for distribution experiments of TEMPO(BF$_4$) and glycerol in a biphasic system

The standard setup for distribution experiments was the two-layered system of gel/aqueous solution ratios of 1:3, 2:3, and 3:3 (in v/v 100 µL) (images of an experimental setup v/v 2:3 in Table S2, Entry 2). The gels differed by either containing **TEMPO(BF$_4$)** (0.1 M), or the aqueous layer contained glycerol (0.03 M, 0.05 M, or 0.1 M). The gel (100 µL, 200 µL, or 300 µL) was added to a vial after which 300 µL of aqueous solution (sat. NaCl, $NaHCO_3$ pH 8.3) was slowly added on top of the gel. As a control, bilayer systems lacking PVDF–HFP were made to analyze the organic layer. After 16 hours, aliquots of the aqueous layer and/or the organic layer were obtained as derivatized as described above, prior to GC analysis to study the distribution of glycerol and **TEMPO(BF$_4$)** over the aqueous and organic phase.

Cleaning of the FTO electrodes

The Fluorine doped Tin Oxide (FTO) electrodes (Solaronix, 2.2 mm, 15 Ω sq^{-1}) were scrubbed with Deconex and rinsed with hot water, followed by wiping with an acetone-soaked tissue and air drying. The FTO electrodes were then rinsed with acetone, toluene, and ethanol and left to air dry in between. The FTO electrodes were then placed inside a glass container in a solution of a teaspoon of Deconex in Milli-Q and sonicated for 30 minutes. The sonication procedure was repeated with Milli-Q, and then ethanol and the electrodes s were left to dry, after which the electrodes were treated with a UV–ozone generator (Ultra-Violet Products, PR-100) for a minimum of 30 minutes.

Preparation of the photoanode FTO|TiO$_2$

A clean FTO plate (Solaronix, 2.2 mm, 15 Ω sq^{-1}) (10×5 cm) was added to a 40 mM $TiCl_4$ solution in Milli-Q (100 mL) and placed in the oven at 70°C for 30 min to create a TiO_2 blocking layer. The FTO plate was consecutively rinsed with Milli-Q, then ethanol and air-dried. The electrodes were sintered with progressive heating (Programmer PR 5) from ambient temperature to 125°C (over 5 min, 25°C min^{-1} ramp, hold 125°C for 5 min) then to 202°C (over 15 min, 5.1°C min^{-1} ramp, hold 202°C for 5 min) to 375°C (over 5 min, 34.6°C min^{-1} ramp, hold 375°C for 5 min) to 450°C (over 5 min, 15°C min^{-1} ramp, hold 450°C for 15 min) to 500°C (over 5 min, 10°C min^{-1} ramp, hold 500°C for 30 min). One mesoporous layer of TiO_2 nanoparticles (particle size 22–25 nm, Dyenamo DN-GPS-22OS, Anatase, Specific surface area 50 - 60 m^2 g^{-1}) was screen printed (43T screen with 0.79 cm^2 circles for DSPECs, 0.2 cm^2 circles for DSSCs) onto the cooled FTO plate thickness of layer after annealing (3.8 µm).[9] The plate was dried for 7 min on a hotplate set at 125°C and cooled to ambient temperature, before printing (43T screen) a scattering layer of TiO_2 (particle size > 100 nm, Solaronix Ti-Nanoxide R/SP, Specific surface area 15 m^2 g^{-1}) thickness of layer after annealing (6.4 µm, Figure S15) and sintered with progressive heating from ambient temperature to 125°C (over 5 min, 25°C min^{-1} ramp, hold 129°C for 5 min) then to 202°C (over 15 min, 5.1°C min^{-1} ramp, hold 202°C for 5 min) to 375°C (over 5 min, 34.6°C min^{-1} ramp, hold 375°C for 5 min) to 450°C (over 5 min, 15°C min^{-1} ramp, hold 450°C for 15 min) to 500° C (over 5 min, 10°C min^{-1} ramp, hold 500°C for 30 min). The cooled sintered plate was soaked for 30 min at 70°C in a 40 mM $TiCl_4$ in Milli-Q and sintered with progressive heating from ambient temperature to 125°C (over 5 min, 25°C min^{-1} ramp, hold 125°C for 5 min) then to 202°C (over 15 min, 5.1°C min^{-1} ramp, hold 202°C for 5 min) to 375°C (over 5 min, 34.6°C min^{-1} ramp, hold 375°C for 5 min) to 450°C (over 5 min, 15°C min^{-1} ramp, hold 450°C for 15 min) to 500° C (over 5 min, 10°C min^{-1} ramp, hold 500°C for 30 min) prior cooling to room temperature for sensitization. The FTO|TiO_2 electrodes were sensitized with 0.5 mM **AP11** in a 1:1 *t*-BuOH/acetonitrile solution in the dark overnight and rinsed afterward with a 1:1 *t*-BuOH/acetonitrile solution and kept in the dark before use. The electrodes were characterized with SEM and showed similar results as in previous projects.[10]

Preparation of the platinum counter electrodes

A clean, with one-hole predrilled (Diamond tip nr. 7134, Dremel-400) FTO plate (Solaronix, 2.2 mm, 15 Ω sq^{-1}) was added to a solution of concentrated 1 M $HCl_{(aq)}$ (37% w/v) in EtOH and sonicated for 30 minutes. The electrodes were rinsed with Milli-Q, EtOH and left to dry. Electrochemical deposition of platinum was performed using an aqueous $PtCl_4$ solution (10 mM $PtCl_4$, 50 mM HCl, 0.47 mM 3-(2-aminoethylamino)propyldimethoxymethylsilane) in a three-electrode system, with the FTO-plate as the working electrode (WE), Ag/AgCl (leakless, eDAQ, ET069) reference electrode (RE) and a Pt-mesh counter electrode (CE). The platinum was deposited onto the FTO- electrodes by chronoamperometry (galvanostatic) employing a set current of -0.025 A vs. Ag/AgCl over 30 seconds with a PGSTAT10 potentiostat (Autolab), yielding a black hue at the FTO-electrodes. Finally, the FTO|Pt electrodes were rinsed with demi water and ethanol and left to air dry before use.

Construction of dye-sensitized solar cells (DSSCs)

Sensitized TiO_2 photoanodes and Pt counter electrodes were sandwiched together using a hotmelt ionomer (60 µm, Meltonix polymer 1170-60, Solaronix, Switzerland) and a soldering iron (320°C, Weller, PU-81) or using Teflon spacers and pressed together with a clothespin-like device (Figure S4). The electrolyte (1.2 M LiTFSI, 1.0 M **TEMPO**, 0.1 M **TEMPO(BF_4)** in 10%wt PVDF–HFP in acetonitrile) was introduced into the cells by vacuum backfilling for 60 µm cells or by (hot) layering onto the photoanode containing the spacer for the thicker cells. For the 60 µm cells, the outside of the cell was thoroughly cleaned by wiping the cell with a tissue moistened with EtOH before sealing with more Meltonix and a glass coverslip, using a soldering iron (320°C) to melt the polymer. The DSSCs were measured masked (0.07 cm^2) by using a P211 potentiostat (Zahner TLS3, 100 mW cm^{-1} LED lamp).

Illumination setup for of the dye-sensitized photoelectrochemical cells (DSPECs)

A three-electrode DSPEC system was composed of two compartments (working and counter) separated by a Nafion-117 membrane (FuelCellStore). WE compartment consists of an FTO|TiO_2|**AP11** photoanode overlayed with a 1.0 M **TEMPO** 3 mm redox-gel (10%wt PVDF–HFP, 1.2 M LiTFSI in acetonitrile) and filled with 0.1 M glycerol in an aqueous solution (sat. NaCl, NaHCO3 pH 8.3, 3 mL), and an Ag/AgCl (leakless, eDAQ, ET069) RE which was placed close to the photoanode. The CE compartment contained an electrodeposited FTO|Pt electrode and was filled with 1.0 M AcOH in acetonitrile (3 mL). Preliminary experiments using a catholyte of 1.0 M AcOH in water instead of MeCN afforded similar results in photocurrents during applied bias chopped-light measurements. However, since we have demonstrated H_2 production in MeCN with 1.0 M AcOH in our previous studies,[10,11] continued the use of a MeCN-based catholyte as improving the photoanode environment is the main focus of this work. Chronoamperometric and chopped-light measurements were performed on a P211 potentiostat (Zahner) without stirring for 23 hours while illuminating the photoanode (illuminated area 0.64 cm^2) using a LED white-light source (Zahner, TLS3, 100 mW cm^{-2}). A bias potential of 0.1 *vs*. Ag/AgCl was applied on the WE (P211 potentiostat, Zahner), and determined using preliminary chopped-light experiments, which can be found in Figure S7. To monitor the reaction, aliquots of the aqueous layer were obtained and were TMS-derivatized and analyzed with GC as described before. The integration of half the photocurrent determines the number of electrons to account for two electrons needed per oxidation reaction. The conversion can be enhanced a prolonging the illumination time. However, it is noteworthy that extending the reaction time can cause over-oxidation of the products with sufficient oxidized **TEMPO** present. Examples of glycerol to ketomalonic acid are known using **TEMPO⁺** as an oxidant.[12] There seem to be some photocurrent fluctuations before a steady state of 0.2 mA cm^{-2} is reached (Figure S7B, S10, and S12A). In all cases, an initial spike in photocurrent production is seen in the first 10 to 30 minutes indicating more **TEMPO** present in the TiO_2 pores. After this spike, a drop in photocurrent is observed, indicating less **TEMPO** present since **TEMPO$^+$** is generated. To ensure efficient photocycles, **TEMPO$^+$** must convert back to **TEMPO** by the oxidation reaction of glycerol to glyceraldehyde at the gel-aqueous interface. We surmise that our (gel-based) biphasic DSPECs are very sensitive to diffusional phenomena such as slight stirring of the aqueous bulk electrolyte by external movement due to lab conditions, influencing the rate of the oxidation reaction at the gel-aqueous interface. Some of these issues may be resolved by controlling these movements by *e.g.*, using a flow reactor system. Furthermore, the diffusional limitation within the gel can be battled by further optimization of the gel layer in terms of thickness, gel design, and TiO_2 pore filling.

Quantification of water in redox-gel.

10 mg of redox-gel unused and used in DSPEC were added to 1 mL of freshly distilled dry toluene. The dry toluene was allowed to take up water from the redox gel over the course of 3 hours. The water in the toluene was then determined by using the Karl Fisher technique on an 831 KF Coulometer (Metrohm) with Hydranal solution.

TEMPO$^{0/+}$, glycerol, and glyceraldehyde exchange and product conversion between (organic)gel layer and aqueous layer.

The desirable separation of the organic and aqueous phase and the gel stability in a mixed system were verified, both needed for long-term photoanode protection. The two-phased system continued to be well separated, and the gel stayed intact over multiple days, as seen in Table S2. The distribution of the compounds throughout the biphasic system is examined next and shown in Table S1. A high affinity of the redox-mediating catalyst **TEMPO$^{0/+}$** for the redox-gel layer is of great importance since sufficient amounts of the redox mediator near the photoanode is necessary for DSPEC to complete photocycles. In contrast, preference of the product and the substrate for the aqueous layer are desired for product retrievement. **TEMPO$^{0/+}$** shows a high affinity (~95%) for the acetonitrile-gel throughout different layer thicknesses due to the over-saturated aqueous solution, while both glycerol (~85%) and glyceraldehyde (97%) prefer the aqueous layer. The strong separation of **TEMPO^{+}** to glycerol in the gel–aqueous system can influence the oxidation reaction considering the two compounds can only react with each other at the interface. Substrate conversion was studied in a biphasic system and compared to a thoroughly mixed reaction mixture. **TEMPO(BF_4)**[2] mimicked the role of *in situ* photogenerated **TEMPO^{+}** in the photosynthesis cell and was used as a chemical oxidant for glycerol. The gel consisted of 10%wt PVDF–HFP in acetonitrile and contained the one-electron **TEMPO(BF_4)** (~1.5 eq.) while glycerol (~1 eq.) was added to the aqueous solution (sat. NaCl, $NaHCO_3$ pH 8.3), which was layered in a 1-to-1 fashion on the acetonitrile-gel. The biphasic system was left to react for 16 hours, after which the two-electron oxidation was monitored, and samples of the organic and aqueous phase were taken at *t*=16 hours, TMS-derivatized and analyzed with gas chromatography (GC)[4,8] (Table S2–5). The formation of glyceraldehyde and the distribution of the substrate, product, and **TEMPO$^{0/+}$** over the gel–aqueous system are quantified, giving the main results in Table 1. A conversion of ~17% of glycerol to glyceraldehyde is seen in the biphasic system and only half of the conversion of 30% in a fully mixed system.[13] The decrease in conversion indicates diffusion limitation plays a role in the oxidation reaction during 16 hours. Considering the presence of the oxidation reaction and the affinity of **TEMPO** to the gel-layer and the product and substrate to the aqueous layer, we believe that this gel is suitable for a biphasic DSPEC system.

Tables and Figures

Table S1: Determined gas chromatography retention times for all used compounds after the TMS derivatization procedure. Analysis was performed on a Trace GC Ultra machine (Interscience) with an RTX-1 column (30 m, 0.25 mm internal diameter, 0.25 µm film thickness), inlet temperature 70°C hold 2.00 minutes ramp 10°C min^{-1} to 340°C. The quantification of the pure glyceraldehyde gave two peaks with retention times 11.3 and 11.7 (min). The two peaks were analyzed with GC–MS, after which we surmise the peaks to correspond to the TMS-protected keto- (Glyceraldehyde_A) and enol- (Glyceraldehyde_B) tautomers of glyceraldehyde. A calibration approach involving the sum of the peak areas was used.[8] However, other possibilities such as the incomplete TMS-derivatization, acetals, monomers, and dimers (through hemiacetal and hemiketal bond formation) of glyceraldehyde can also be suitable as the exact species is often hard to determine, as discussed by A. Parodi, E. Diguilio, S. Renzini, I. Magario in *Carbohydrate Research* **2020**, *487*, 107885. Since the qualification on the TMS-glyceraldehyde species formed during the aqueous derivatization process is generally very strenuous, we would like to refer the reader to the previous mentioned reference for more information and in-depth discussion on the GC analysis of these compounds.[8]

Chemical compound	Determined retention time (min)
Glycerol	9.914
Glyceraldehyde (A)	11.317
Glyceraldehyde (B)	11.679
Dihydroxyacetone	8.940
1-hexanol	5.617
(2,2,6,6-Tetramethylpiperidin-1-yl)oxyl$^{0/+}$	7.561

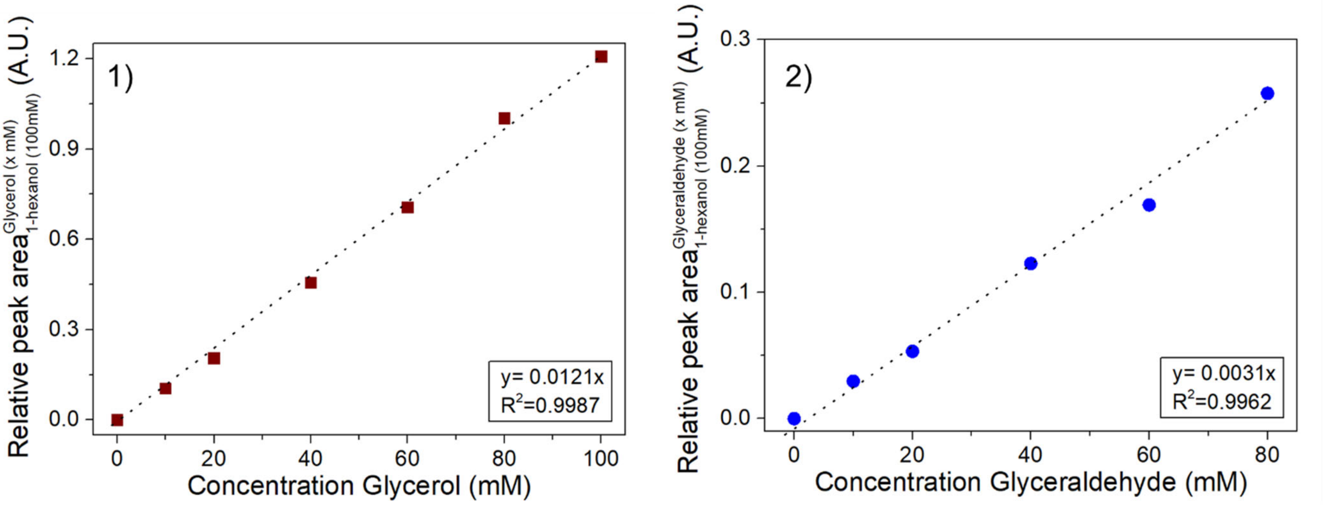

Figure S1,2. Gas chromatography calibration curve for 1) glycerol and 2) glyceraldehyde in aqueous solution (sat. NaCl, $NaHCO_3$ pH 8.3) relative to 1-hexanol (100 mM) as external standard after the TMS derivatization procedure. Analysis was performed on a Trace GC Ultra machine (Interscience) with an RTX-1 column (30 m, 0.25 mm internal diameter, 0.25 µm film thickness), inlet temperature 70°C hold 2.00 minutes ramp 10°C min^{-1} to 340°C.

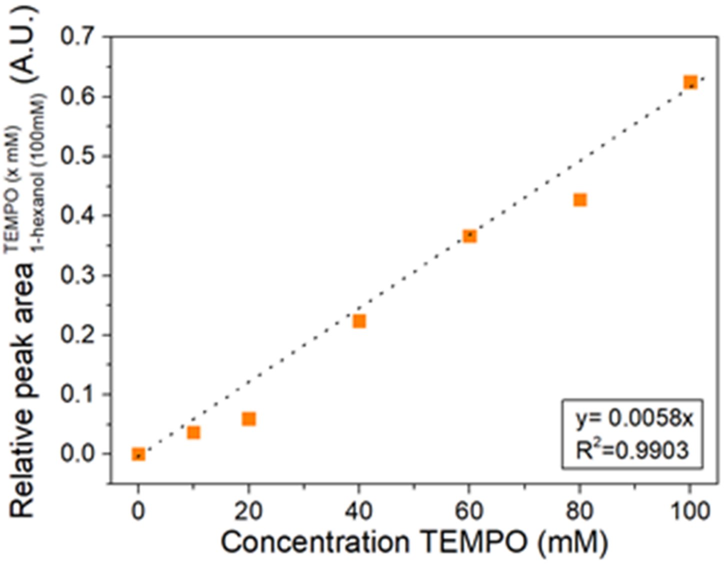

Figure S3. Gas chromatography calibration curve for **TEMPO(BF$_4$)** in aqueous solution (sat. NaCl, $NaHCO_3$ pH 8.3) relative to 1-hexanol (100 mM) as external standard after the TMS derivatization procedure. Analysis was performed on a Trace GC Ultra machine (Interscience) with an RTX-1 column (30 m, 0.25 mm internal diameter, 0.25 µm film thickness), inlet temperature 70°C hold 2.00 minutes ramp 10°C min^{-1} to 340°C.

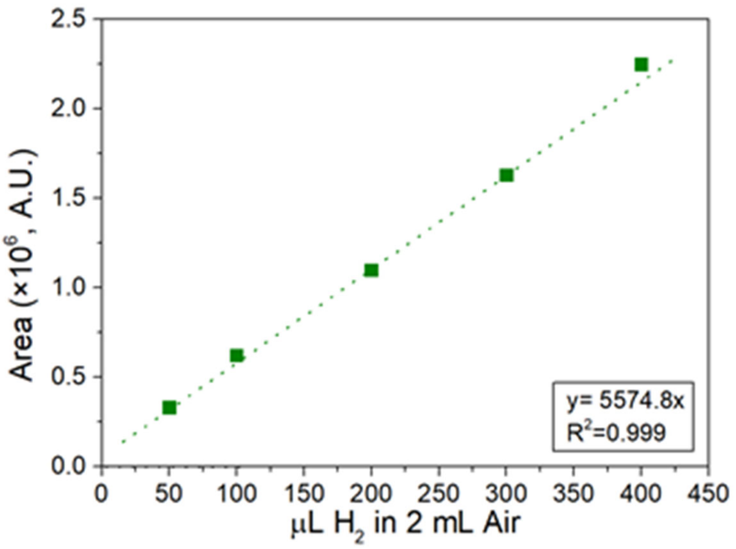

Figure S4. Gas chromatography calibration curve for H_2 in 2 mL air (1 mol of H_2 = 22.4 L of H_2). Hydrogen qualification was measured on a Gas Chromatograph (Shimadzu Nexis GC-2030) with a 5Å mol sieve column (60 m, 0.32 mm internal diameter, 25 µm film thickness) with an inlet temperature of 40°C and a flow argon rate of 8.0 mL min^{-1}.

Table S2. Overview of setup for chemical oxidation reactions with **TEMPO(BF_4)**. In gel biphasic systems: the gel layer consists of 10%wt PVDF–HFP, 1.2 M LiTFSI in acetonitrile, and the aqueous layer is sat. NaCl, $NaHCO_3$ pH 8.3 in H_2O. In acetonitrile biphasic systems: the organic layer consist of 1.2 M LiTFSI in acetonitrile, and the aqueous layer is sat. $NaHCO_3$ pH 8.3 in H_2O. A fully mixed system consists of acetonitrile in $NaHCO_3$ pH 8.3 in H_2O. The gel/acetonitrile layer is on the bottom at *t*=0 hours but moves to the top at *t*=16 hours due to the lower density of the layer and the low friction of the glass vial.

Entry	Image reaction vial	Description
1	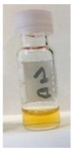	Fully mixed system:<br>0.2 M **TEMPO(BF_4)** in 200 μL acetonitrile and 0.2 M glycerol in 200 μL aqueous sat. $NaHCO_3$ pH 8.3
2	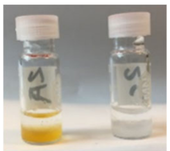	(2:3) Bilayed system for diffusion<br>Left: (bottom) 200 μL gel layer 0.1 M **TEMPO(BF_4)**, (top) 300 μL aqueous layer<br>Right: (bottom) 200 μL gel layer, (top) 300 μL aqueous layer 0.1 M glycerol<br>((gel or acetonitrile: aq) (100 μL: 300 μL) and (300 μL : 300 μL) are executed similar)
3	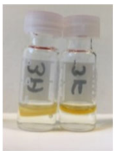	(1:3) Bilayed system for chemical oxidation<br>Left: (top) 100 μL gel layer 0.1 M **TEMPO(BF_4)**, (bottom) 300 μL aqueous layer 0.033 M glycerol<br>Right: (top) 100 μL acetonitrile layer 0.1 M **TEMPO(BF_4)**, (bottom) 300 μL aqueous layer 0.033 M glycerol
4	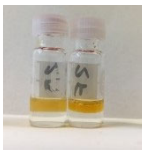	(2:3) Bilayed system for chemical oxidation<br>Left: (top) 200 μL gel layer 0.1 M **TEMPO(BF_4)**, (bottom) 300 μL aqueous layer 0.066 M glycerol<br>Right: (top) 200 μL acetonitrile layer 0.1 M **TEMPO(BF_4)**, (bottom) 300 μL aqueous layer 0.066 M glycerol
5	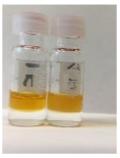	(3:3) Bilayed system for chemical oxidation<br>Left: (top) 300 μL gel layer 0.1 M **TEMPO(BF_4)**, (bottom) 300 μL aqueous layer 0.1 M glycerol<br>Right: (top) 300 μL acetonitrile layer 0.1 M **TEMPO(BF_4)**, (bottom) 300 μL aqueous layer 0.1 M glycerol

Table S3 Overview chemical oxidation reactions of **TEMPO(BF$_4$)** and glycerol in gel biphasic systems: the gel layer consists of 10%wt PVDF–HFP, 1.2 M LiTFSI in acetonitrile, and the aqueous layer is sat. NaCl, $NaHCO_3$ pH 8.3 in H_2O. In acetonitrile biphasic systems: the organic layer consist of 1.2 M LiTFSI in acetonitrile, and the aqueous layer is sat. $NaHCO_3$ pH 8.3 in H_2O. Glycerol was added to the aqueous layer and **TEMPO(BF$_4$)** at the acetonitrile layer or gel *at t*=0 hours. Ratios of glycerol and **TEMPO(BF4)** are equal in all systems (1:1.5). The reaction was done overnight at room temperature Data is an average of *N*=2. GC analysis was performed after the TMS derivatization procedure on a Trace GC Ultra machine (Interscience) with an RTX-1 column (30 m, 0.25 mm internal diameter, 0.25 µm film thickness), inlet temperature 70°C hold 2.00 minutes ramp 10°C min^{-1} to 340°C.

Entry	Layer measured	Layer size	**TEMPO** *t=0* hours			Glycerol *t=0* hours			Glyceraldehyde *t=0* hours			**TEMPO** *t=16* hours			Glycerol *t=16* hours			Glyceraldehyde *t*=16 hours			Conversion (%) glycerol to glyceraldehyde		
		(mL)	mM	µmol	%	mM	µmol	%	mM	µmol	%	mM	µmol	%	mM	µmol	%	(mM)	µmol	%	Raw	Max	Calc
1A	Acetonitrile	0.3	226.5	68.0	100.0	0.0	0.0	0.0	0.0	0.0	-	219.2	65.8	96.8	16.9	5.1	14.8	2.4	0.7	12.9	1.8		2.1
	Aqueous	0.3	0.0	0.0	0.0	138.0	41.4	100	0.0	0.0	-	7.3	2.2	3.2	97.0	29.1	85.2	16.2	4.9	87.1	12.0		14.3
	Total	0.6	226.5	68.0	100.0	138.0	41.4	100.0	0.0	0.0	-	226.5	68.0	100.0	114.0	34.2	100.0	18.6	5.6	100.0	13.8	81.9	16.4
1B	Gel	0.3	-	-	-	-	-	-	-	-	-	-	-	-	-	-	-	-	-	-	-	-	-
	Aqueous	0.3	0.0	0.0	0.0	138.0	41.4	100.0	0.0	0.0	-	7.7	2.3	-	97.4	29.2	-	18.9	5.7	-	14.0	-	16.7
	Total	0.6	-	-	-	-	-	-	-	-	-	-	-	-	-	-	-	-	-	-	-	-	-
2A	Acetonitrile	0.2	232.1	46.9	100.0	0.0	0.0	0.0	0.0	0.0	-	226.8	45.4	96.7	17.2	3.4	14.0	5.2	1.0	14.8	5.2		6.8
	Aqueous	0.3	0.0	0.0	0.0	99.6	29.9	100.0	0.0	0.0	-	5.2	1.6	3.3	70.2	21.1	86.0	19.8	6.0	85.2	20.0		26.3
	Total	0.5	232.1	46.9	100.0	99.6	29.9	100.0	0.0	0.0	-	232.1	46.9	100.0	87.4	24.5	100.0	25.0	7.0	100.0	25.3	75.7	33.2
2B	Gel Layer	0.2	-	-	-	-	-	-	-	-	-	-	-	-	-	-	-	-	-	-	-	-	-
	Aqueous	0.3	0.0	0.0	0.0	99.6	29.9	100.0	0.0	0.0	-	5.5	1.7	-	75.6	22.7	-	19.9	6.0	-	20.1	-	26.4
	Total	0.6	-	-	-	-	-	-	-	-	-	-	-	-	-	-	-	-	-	-	-	-	-
3A	Acetonitrile	0.1	276.5	29.3	100.0	0.0	0.0	0.0	0.0	0.0	-	268.4	26.8	91.7	9.7	1.0	9.2	0.3	0.0	1.6	0.6		0.7
	Aqueous	0.3	0.0	0.0	0.0	48.5	14.6	100.0	0.0	0.0	-	8.1	2.4	8.3	31.8	9.5	90.8	6.1	1.8	98.4	12.3		14.1
	Total	0.4	276.5	29.3	100.0	48.5	14.6	100.0	0.0	0.0	-	276.5	29.3	100.0	41.5	10.5	100.0	6.4	1.9	100.0	12.9	89.9	14.7
3B	Gel	0.1	-	-	-	-	-	-	-	-	-	-	-	-	-	-	-	-	-	-	-	-	-
	Aqueous	0.3	0.0	0.0	0.0	48.5	14.6	100.0	0.0	0.0	-	5.5	1.7	-	34.3	10.3	-	4.6	0.6	-	9.2	-	10.6
	Total	0.4	-	-	-	-	-	-	-	-	-	-	-	-	-	-	-	-	-	-	-	-	-
4	Fully mixed system	0.4	154.3	61.7	100.0	100.0	40.0	100.0	0.0	0.0	-	154.3	61.7	-	44.8	17.9	-	20.8	8.3		20.8	77.1	27.0

Table S4 Overview of biphasic exchange studies of **TEMPO(BF_4)**. In gel biphasic systems: the gel layer consists of 10%wt PVDF–HFP, 1.2 M LiTFSI in acetonitrile, and the aqueous layer is sat. NaCl, $NaHCO_3$ pH 8.3 in H_2O. In acetonitrile biphasic systems: the organic layer consist of 1.2 M LiTFSI in acetonitrile, and the aqueous layer is sat. $NaHCO_3$ pH 8.3 in H_2O. The exchange experiment was done overnight at room temperature. **TEMPO(BF_4)** was added to the acetonitrile of the gel layer *at t*=0 hours. Data is average of *N*=2. * based on the amount added. GC analysis was performed after the TMS derivatization procedure on a Trace GC Ultra machine (Interscience) with an RTX-1 column (30 m, 0.25 mm internal diameter, 0.25 µm film thickness), inlet temperature 70°C hold 2.00 minutes ramp 10°C min^{-1} to 340°C

Entry	Layer measured	Layer size (mL)	**TEMPO** *t*=0 hours (mM)	**TEMPO** *t*=16 hours (mM)	**TEMPO** *t*=0 hours (µmol)	**TEMPO** *t*=16 hours (µmol)	**TEMPO** *t*=0 hours (%)	**TEMPO** *t*=16 hours (%)
1A	Acetonitrile	0.3	205.6	196.8	61.7	59.1	100.0	95.7
	Aqueous	0.3	0	8.7	0.0	2.6	0.0	4.2
1B	Gel	0.3	-	-	-	-	-	-
	Aqueous	0.3	0.0	5.8	0.0	1.7	0.0	2.8*
2A	Acetonitrile	0.2	231.5	222.8	46.3	44.6	100.0	96.2
	Aqueous	0.3	0	5.8	0.0	1.7	0.0	3.8
2B	Gel	0.2	-	-	-	-	-	-
	Aqueous	0.3	0.0	5.5	0.0	1.6	0.0	3.5*
3A	Acetonitrile	0.1	292.0	264.5	29.2	26.4	100.0	90.6
	Aqueous	0.3	0	9.2	0.0	2.7	0.0	9.4
3B	Gel	0.1	-	-	-	-	-	-
	Aqueous	0.3	0.0	9.7	0.0	2.1	0.0	7.1*

SUPPORTING INFORMATION

Table S5 Overview of biphasic exchange studies of glycerol in gel biphasic systems: the gel layer consists of 10%$_{WT}$ PVDF–HFP, 1.2 M LiTFSI in acetonitrile, and the aqueous layer is sat. NaCl, $NaHCO_3$ pH 8.3 in H_2O. In acetonitrile biphasic systems: the organic layer consist of 1.2 M LiTFSI in acetonitrile, and the aqueous layer is sat. $NaHCO_3$ pH 8.3 in H_2O. Glycerol was added to the aqueous layer *at t*=0 hours. The exchange experiment was done overnight at room temperature Data is an average of *N*=2. GC analysis was performed after the TMS derivatization procedure on a Trace GC Ultra machine (Interscience) with an RTX-1 column (30 m, 0.25 mm internal diameter, 0.25 μm film thickness), inlet temperature 70°C hold 2.00 minutes ramp 10°C min^{-1} to 340°C

Entry	Layer measured	Layer size (mL)	Glycerol *t*=0 hours (mM)	Glycerol *t*=16 hours (mM)	Glycerol *t*=0 hours (μmol)	Glycerol *t*=16 hours (μmol)	Glycerol *t*=0 hours (%)	Glycerol *t*=16 hours (%)
1A	Acetonitrile	0.3	0.0	22.0	0.0	6.6	0.0	16.5
	Aqueous	0.3	133.3	111.3	40.0	33.4	100.0	83.5
1B	Gel	0.3	-	-	-	-	-	-
	Aqueous	0.3	133.3	125.2	40.0	37.5	100.0	93.9
2A	Acetonitrile	0.2	0.0	20.0	0.0	4.0	0.0	12.9
	Aqueous	0.3	122.2	90.0	31.0	27.0	100.0	87.1
2B	Gel	0.2	-	-	-	-	-	-
	Aqueous	0.3	122.2	94.7	31.0	28.4	100.0	91.6
3A	Acetonitrile	0.1	0.0	14.9	0.0	1.5	0.0	11.1
	Aqueous	0.3	54.7	39.8	13.4	11.9	100.0	88.9
3B	Gel	0.1	-	-	-	-	-	-
	Aqueous	0.3	54.7	42.1	13.4	12.6	100.0	94.1

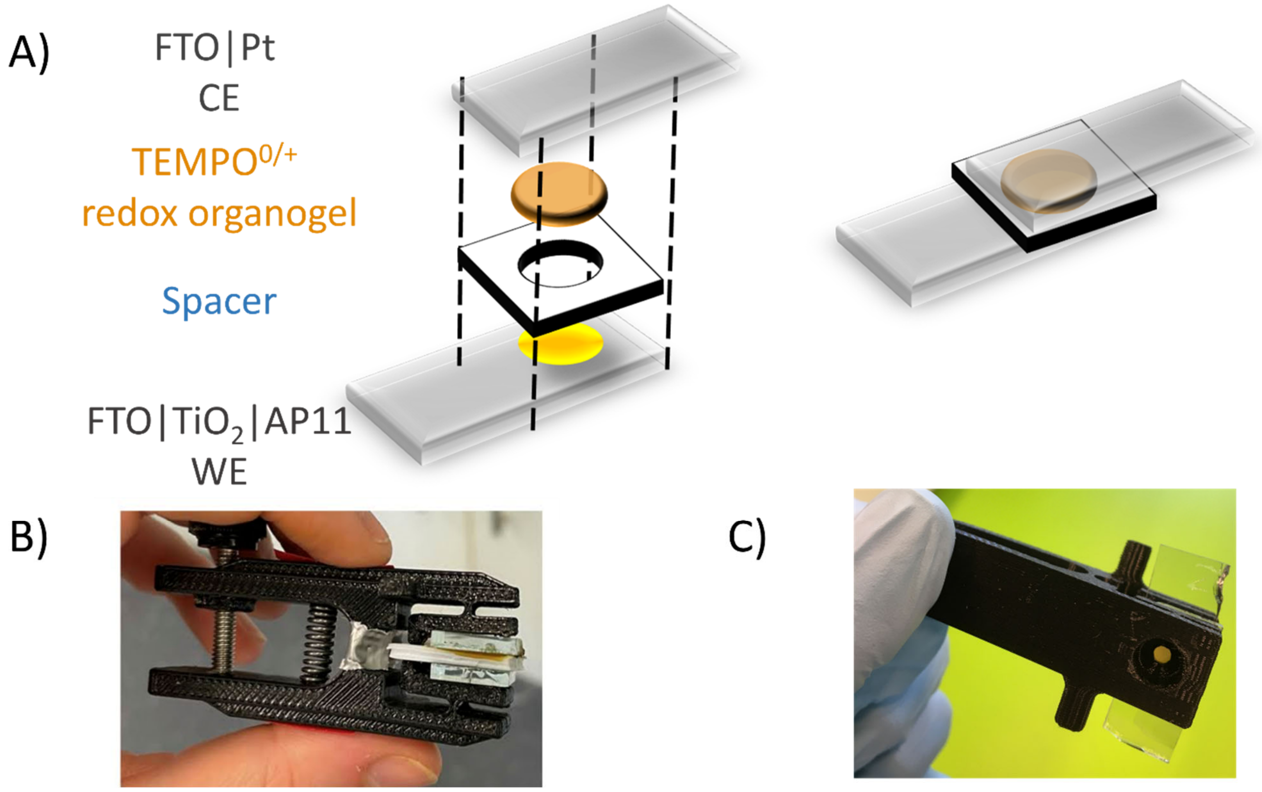

Figure S5. A) Schematic overview of the assembly of a DSSC with **TEMPO**$^{+/0}$ redox-gel as quasi-solid state electrolyte. Redox-gel was sandwiched between an FTO|TiO$_2$|**AP11** working electrode and an FTO|Pt (electrodeposited) counter electrode. The correct thickness of the redox-gel was controlled with a Teflon spacer. **B)** Side-view of the Teflon clothespin device, including a mechanism to keep DSSC perpendicular to the incoming light source. **C)** Top-view of the clothespin device, including a mask (3 mm diameter), which controlled the illuminated area of the photoanode.

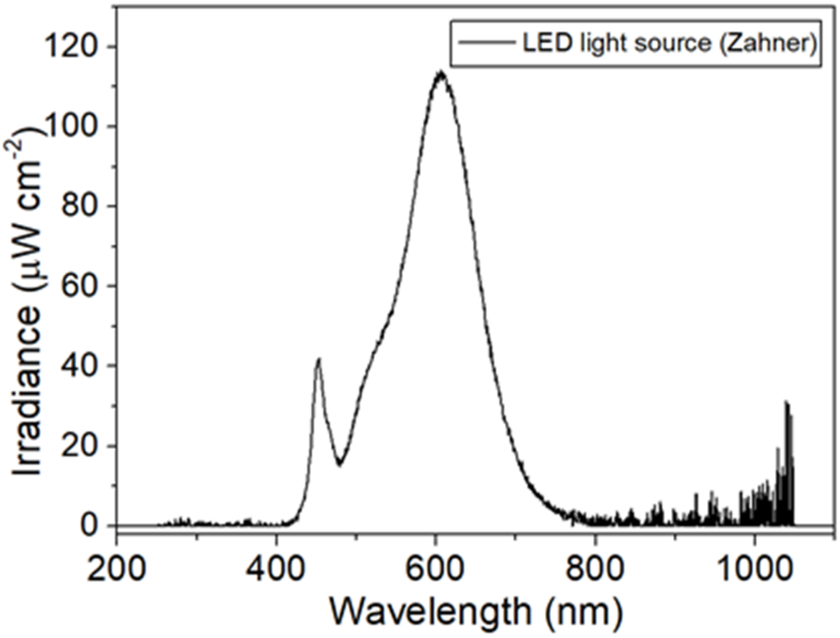

Figure S6. Reference spectra of used illumination source: LED white-light source (Zahner, TLS3, 50 mW cm^{-2}).

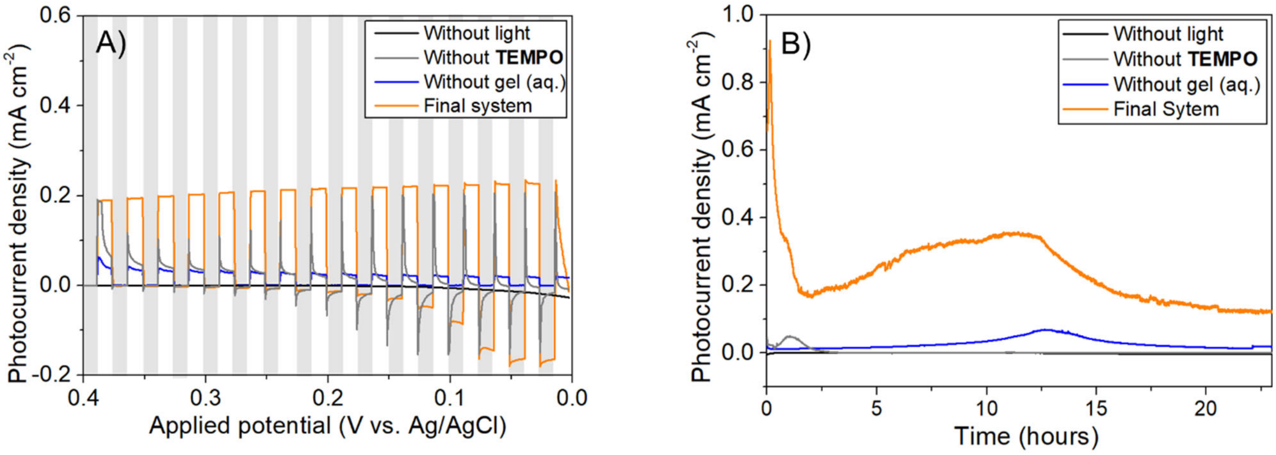

Figure S7. **A)** Initial chopped light experiments of the various DSPEC systems with potential was scanned from 0.0 V to 0.40 V *vs*. Ag/AgCl**. B)** Overview of the photogenerated current density in all DSPEC measurements over a period of 24 hours with an applied bias potential of 0.1 *vs*. Ag/AgCl on the WE (P211 potentiostat, Zahner). Final system (unless otherwise noted): WE electrode compartment consisted of an FTO|TiO_2|**AP11** WE overlayed with a 1.0 M **TEMPO** 3 mm redox-gel (10%wt PVDF–HFP, 1.2 M LiTFSI in acetonitrile) and filled with 0.1 M glycerol aqueous solution (sat. NaCl, $NaHCO_3$ pH 8.3, 3 mL). An Ag/AgCl RE which was placed close to the WE. The CE compartment was separated by a Nafion-117 membrane and consisted of a FTO|Pt CE and was filled with 1.0 M AcOH in acetonitrile (3 mL) and illuminated (masked size 0.64 cm^2) with a LED light source (Zahner, TLS3, 100 mW cm^{-2}) with grey area=light off and white area= light on. Chopped-light and chronoamperometric measurements were performed on a P211 potentiostat (Zahner).

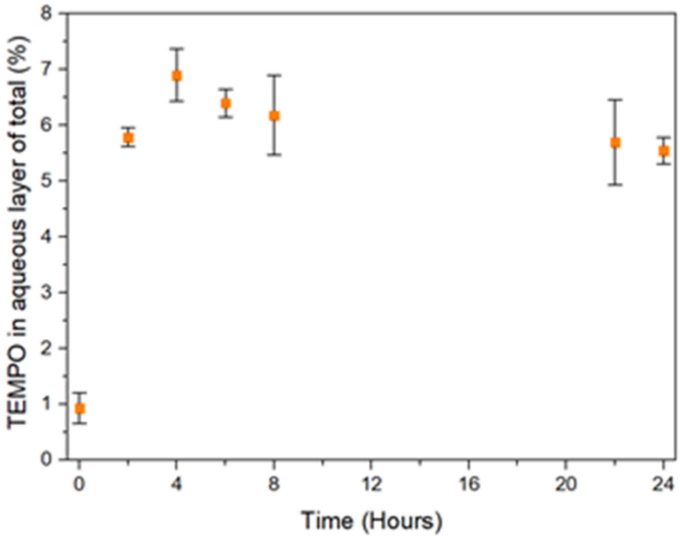

Figure S8. Graph of **TEMPO** present in the aqueous layer in DSPEC experiments obtained over 24 hours. WE electrode compartment consisted of an FTO|TiO_2|**AP11** WE overlayed with a 1.0 M **TEMPO** 3 mm redox-gel (10%wt PVDF–HFP, 1.2 M LiTFSI in acetonitrile) and filled with 0.1 M glycerol in an aqueous solution (sat. NaCl, $NaHCO_3$ pH 8.3, 3 mL). An Ag/AgCl RE was placed close to the WE. The CE compartment was separated by a Nafion-117 membrane and consisted of an FTO|Pt CE and was filled with 1.0 M AcOH in acetonitrile (3 mL). A bias potential of 0.1 *vs*. Ag/AgCl was applied on the WE (P211 potentiostat, Zahner) and the system was illuminated with a LED light source (Zahner, TLS3, 100 mW cm^{-2}) (masked size 0.64 cm^2). GC analysis was performed on samples of the aqueous reaction mixture, after the TMS derivatization procedure, on a Trace GC Ultra machine (Interscience) with an RTX-1 column (30 m, 0.25 mm internal diameter, 0.25 μm film thickness), inlet temperature 70°C hold 2.00 minutes ramp 10°C min^{-1} to 340°C

Table S6. Increase in water content of gel during 48 hours DSPEC measurements determined with the Karl Fisher technique on an 831 KF Coulometer (Metrohm) with Hydranal solution. The 1.0 M **TEMPO** 3 mm redox-gel (10%wt PVDF–HFP, 1.2 M LiTFSI in acetonitrile) was soaked in a 0.1 M glycerol aqueous solution (sat. NaCl, $NaHCO_3$ pH 8.3, 3 mL).

Entry	Experiment	H_2O in 1 mL Toluene (ppm, *N*=3, (σ))	H_2O in 1 mL Toluene (μg)	H_2O in $Toluene_{end-start}$ (μg)	H_2O in gel (per 10 mg gel, %)	Increase H_2O content in gel (%)
1	Dry Toluene (1 mL)	41.0 (8.5)	47	0.00	0.00	
2	Unused gel (10 mg Gel in 1 mL)	249.4 (40.6)	290	240	2.4	
3	Used Gel (10 mg Gel in 1 mL)	394.3 (80.2)	450	410	4.1	1.7

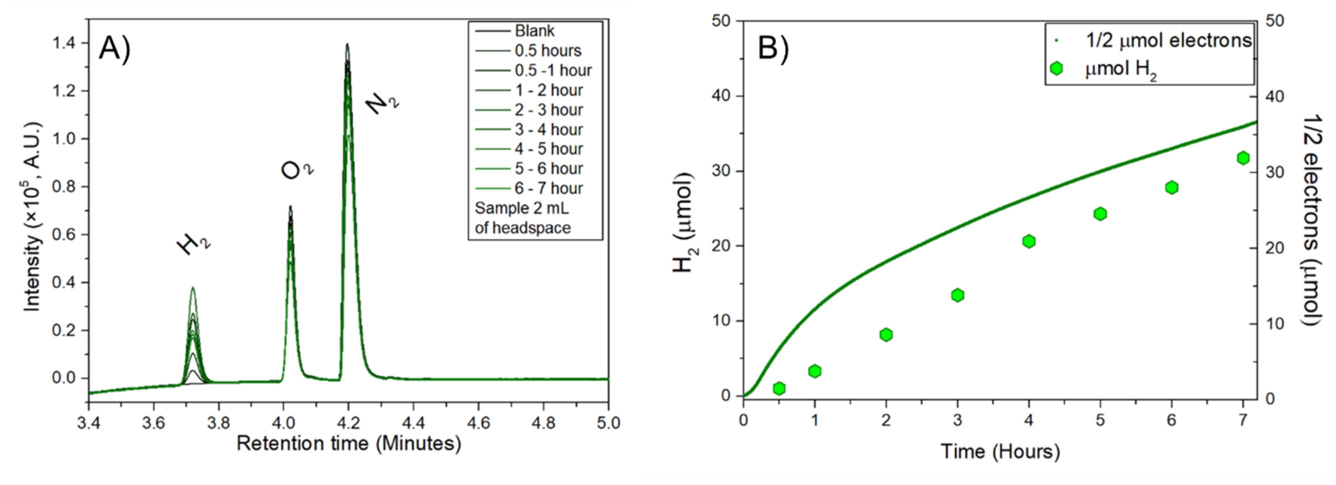

Figure S9. A) H_2 measurement in DSPEC experiments and **B)** H_2 and 1/2 electrons produced in DSPEC experiments over a period of 7 hours. Gas Chromatograph (Shimadzu Nexis GC-2030) with a 5Å mol sieve column (60 m, 0.32 mm internal diameter, 25 µm film thickness) with an inlet temperature of 40°C and a flow argon rate of 8.0 mL min^{-1}. WE electrode compartment consisted of an FTO|TiO_2|**AP11** WE overlayed with a 1.0 M **TEMPO** 3 mm redox-gel (10%wt PVDF–HFP, 1.2 M LiTFSI in acetonitrile) and filled with 0.1 M glycerol in an aqueous solution (sat. NaCl, $NaHCO_3$ pH 8.3, 3 mL). An Ag/AgCl RE was placed close to the WE. The CE compartment was separated by a Nafion-117 membrane and consisted of an FTO|Pt CE and was filled with 1.0 M AcOH in acetonitrile (3 mL). A bias potential of 0.1 *vs*. Ag/AgCl was applied on the WE (P211 potentiostat, Zahner) and the system was illuminated with a LED light source (Zahner, TLS3, 100 mW cm^{-2}) (masked size 0.64 cm^2). Headspace (2 mL) of the counter electrode compartment was sampled. Chronoamperometric measurements were performed on a P211 potentiostat (Zahner).

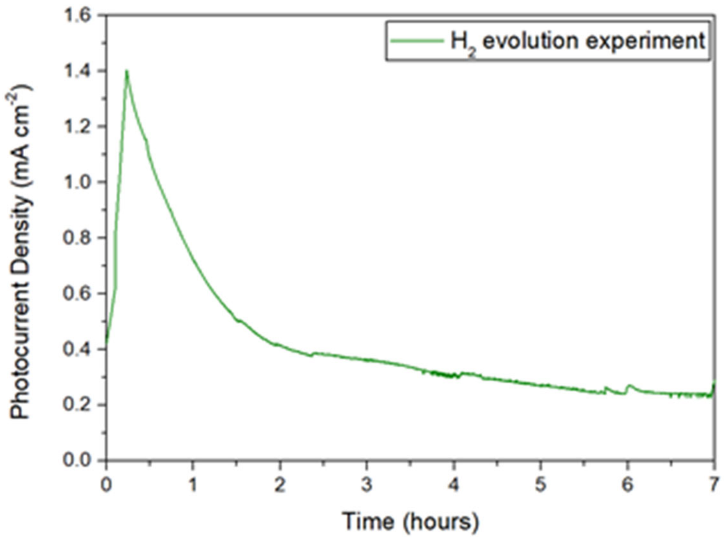

Figure S10. Overview of the photogenerated current density in H_2 DSPEC measurements over a period of 7 hours. WE electrode compartment consisted of an FTO|TiO_2|**AP11** WE overlayed with a 1.0 M **TEMPO** 3 mm redox-gel (10%wt PVDF–HFP, 1.2 M LiTFSI in acetonitrile) and filled with 0.1 M glycerol in an aqueous solution (sat. NaCl, $NaHCO_3$ pH 8.3, 3 mL). An Ag/AgCl RE was placed close to the WE. The CE compartment was separated by a Nafion-117 membrane and consisted of an FTO|Pt CE and was filled with 1.0 M AcOH in acetonitrile (3 mL). A bias potential of 0.1 *vs*. Ag/AgCl was applied on the WE (P211 potentiostat, Zahner) and the system was illuminated with a LED light source (Zahner, TLS3, 100 mW cm^{-2}) (masked size 0.64 cm^2). Chronoamperometric measurements were performed on a P211 potentiostat (Zahner).

A)

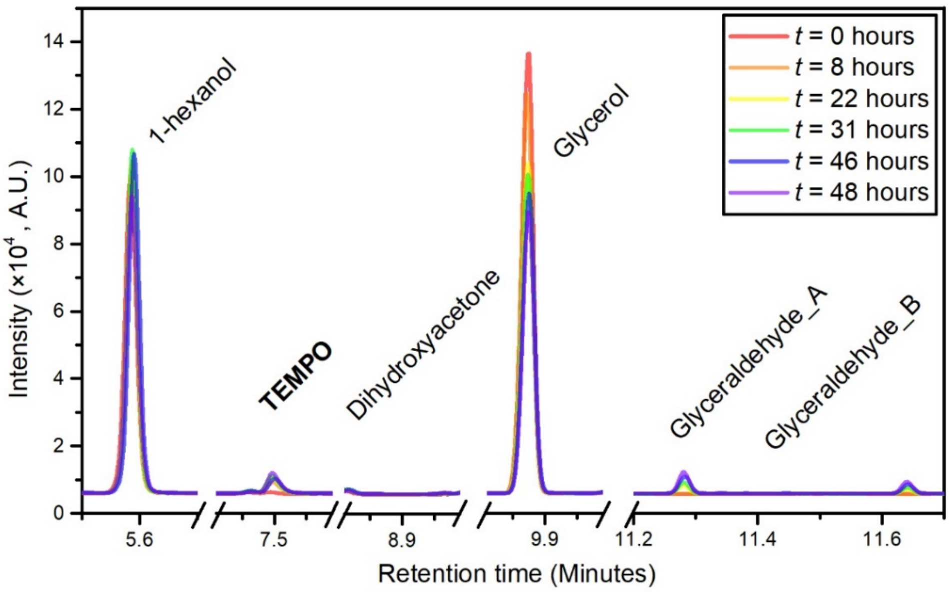

B)

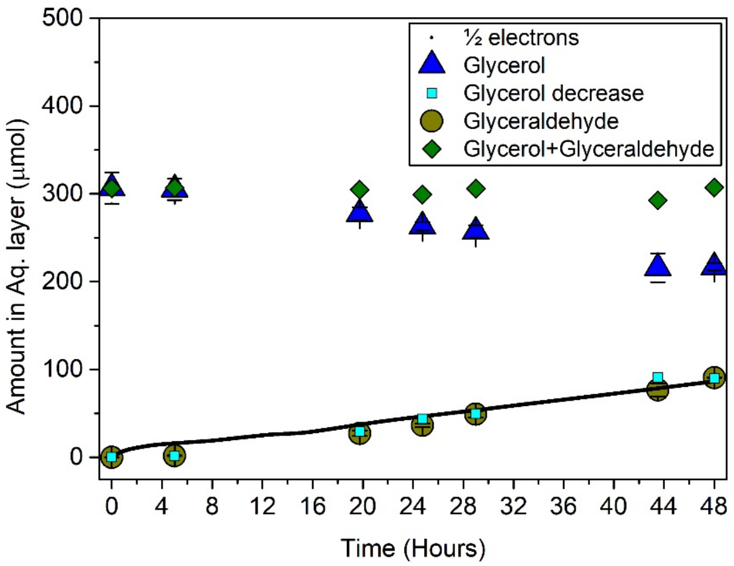

Figure S11. A) GC chromatogram of sampling of the aqueous reaction mixture in long-term DSPEC experiments measured over 48 hours. **B)** Graph of glycerol, decrease in glycerol, glyceraldehyde and total amount of glycerol and glyceraldehyde in the aqueous layer and the corresponding conversion based on photocurrent (in ½ electrons) that were produced in two long-term DSPEC measurements over a period of 48 hours.GC analysis was performed on samples of the aqueous reaction mixture, after the TMS derivatization procedure, on a Trace GC Ultra machine (Interscience) with an RTX-1 column (30 m, 0.25 mm internal diameter, 0.25 µm film thickness), inlet temperature 70°C hold 2.00 minutes ramp 10°C min^{-1} to 340°C. WE electrode compartment consisted of an FTO|TiO_2|**AP11** WE overlayed with a 1.0 M **TEMPO** 3 mm redox-gel (10%wt PVDF–HFP, 1.2 M LiTFSI in acetonitrile) and filled with 0.1 M glycerol in an aqueous solution (sat. NaCl, $NaHCO_3$ pH 8.3, 3 mL). An Ag/AgCl RE was placed close to the WE. The CE compartment was separated by a Nafion-117 membrane and consisted of an FTO|Pt CE and was filled with 1.0 M AcOH in acetonitrile (3 mL). A bias potential of 0.1 *vs.* Ag/AgCl was applied on the WE (P211 potentiostat, Zahner) and the system was illuminated with a LED light source (Zahner, TLS3, 100 mW cm^{-2}) (masked size 0.64 cm^2). The quantification of the pure glyceraldehyde gave the two peaks with retention times 11.3 and 11.7 (min). The two peaks were analyzed with GC–MS, after which we surmised the peaks to correspond to the TMS-protected keto- (Glyceraldehyde_A) and enol- (Glyceraldehyde_B) tautomers of glyceraldehyde. However, other possibilities such as the incomplete TMS-derivatization, acetals, monomers, and dimers (through hemiacetal and hemiketal bond formation) of glyceraldehyde may also be suitable as the exact species is often hard to determine, as discussed by A. Parodi, E. Diguilio, S. Renzini, I. Magario in Carbohydrate Research 2020, 487, 107885. Since the qualification on the TMS-glyceraldehyde species formed during the aqueous derivatization process is generally very strenuous, we would like to refer the reader to the previous mentions reference for more information and in-depth discussion on the GC analysis on these compounds.

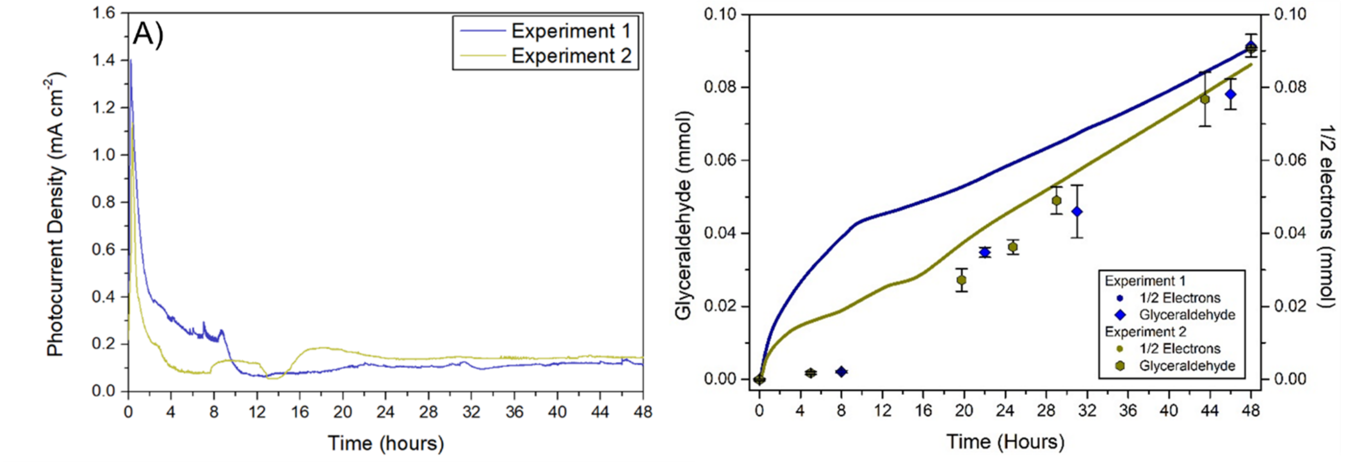

Figure S12. A) Overview of the photogenerated current density produced, and **B)** Graph of glyceraldehyde and 1/2 electrons that were produced in two long-term DSPEC measurements over a period of 48 hours. Final system (unless otherwise noted): WE electrode compartment consisted of an FTO|TiO_2|**AP11** WE overlayed with a 1.0 M **TEMPO** 3 mm redox-gel (10%wt PVDF–HFP, 1.2 M LiTFSI in acetonitrile) and filled with 0.1 M glycerol in an aqueous solution (sat. NaCl, $NaHCO_3$ pH 8.3, 3 mL). An Ag/AgCl RE was placed close to the WE. The CE compartment was separated by a Nafion-117 membrane and consisted of an FTO|Pt CE and was filled with 1.0 M AcOH in acetonitrile (3 mL). A bias potential of 0.1 *vs*. Ag/AgCl was applied on the WE (P211 potentiostat, Zahner) and the system was illuminated with a LED light source (Zahner, TLS3, 100 mW cm^{-2}) (masked size 0.64 cm^2). GC analysis was performed on samples of the aqueous reaction mixture, after the TMS derivatization procedure, on a Trace GC Ultra machine (Interscience) with an RTX-1 column (30 m, 0.25 mm internal diameter, 0.25 µm film thickness), inlet temperature 70°C hold 2.00 minutes ramp 10°C min^{-1} to 340°C. Chronoamperometric measurements were performed on a P211 potentiostat (Zahner).

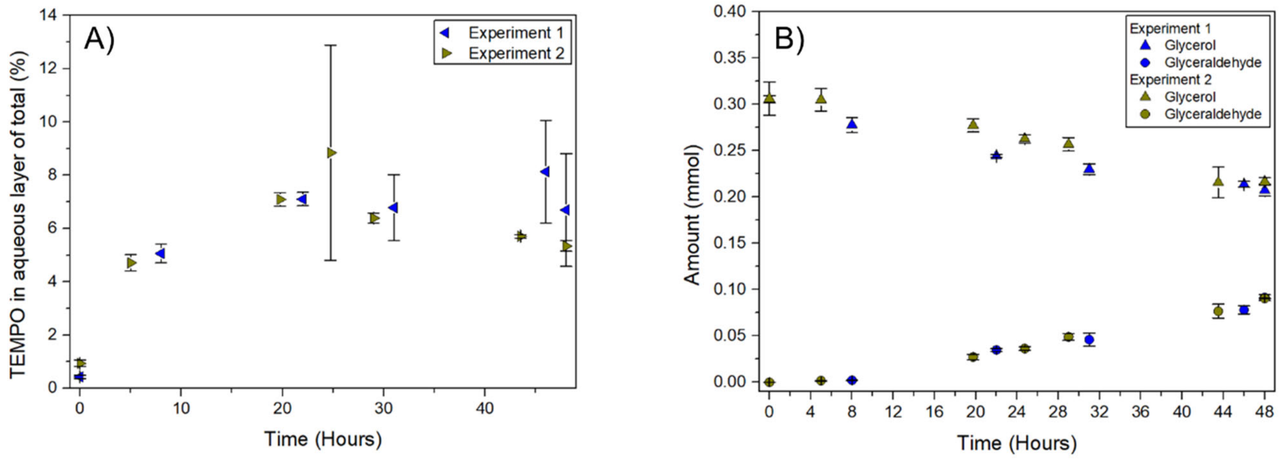

Figure S13. A) TEMPO in the aqueous layer in two long-term DSPEC experiments measured over 48 hours **B)** glycerol and glyceraldehyde in the aqueous layer two long-term DSPEC experiments measured over 48 hours. WE electrode compartment consisted of an FTO|TiO_2|**AP11** WE overlayed with a 1.0 M **TEMPO** 3 mm redox-gel (10%wt PVDF–HFP, 1.2 M LiTFSI in acetonitrile) and filled with 0.1 M glycerol in an aqueous solution (sat. NaCl, $NaHCO_3$ pH 8.3, 3 mL). An Ag/AgCl RE was placed close to the WE. The CE compartment was separated by a Nafion-117 membrane and consisted of an FTO|Pt CE and was filled with 1.0 M AcOH in acetonitrile (3 mL). A bias potential of 0.1 *vs*. Ag/AgCl was applied on the WE (P211 potentiostat, Zahner) and the system was illuminated with a LED light source (Zahner, TLS3, 100 mW cm^{-2}) (masked size 0.64 cm^2). GC analysis was performed on samples of the aqueous reaction mixture, after the TMS derivatization procedure, on a Trace GC Ultra machine (Interscience) with an RTX-1 column (30 m, 0.25 mm internal diameter, 0.25 µm film thickness), inlet temperature 70°C hold 2.00 minutes ramp 10°C min^{-1} to 340°C.

Table S7 Overview of biphasic exchange studies and oxidation studies with **TEMPO(BF_4)** of benzyl alcohol in gel biphasic systems: the gel layer consists of 10%$_{WT}$ PVDF–HFP, 1.2 M LiTFSI in acetonitrile, and the aqueous layer is sat. NaCl, $NaHCO_3$ pH 8.3 in H_2O. In acetonitrile biphasic systems: the organic layer consist of 1.2 M LiTFSI in acetonitrile, and the aqueous layer is sat. $NaHCO_3$ pH 8.3 in H_2O. Benzyl alcohol was added to the aqueous layer *at t*=0 hours. The experiment was done overnight at room temperature. Data is an average of *N*=2. GC analysis was performed on a Trace GC Ultra machine (Interscience) with an RTX-1 column (30 m, 0.25 mm internal diameter, 0.25 µm film thickness), inlet temperature 70°C hold 2.00 minutes ramp 10°C min^{-1} to 340°C.

Entry	Layer measured	Layer size	Benzyl alcohol *t=0* hours			Benzaldehyde *t=0* hours			Benzyl alcohol *t=16* hours			Benzaldehyde *t*=16 hours			Conversion after 16 hours Benzyl alcohol to benzaldehyde (µmol)		
		(mL)	mM	µmol	%	mM	µmol	%	mM	µmol	%	mM	µmol	%	Benzyl alcohol	Benzal dehyde	Conver sion (%)
1	MeCN/ Gel	0.3	0	0	0	0	0	-	20	6	100	78	23.4	97.5			
2	Aqueous	0.3	100	30	100	0	0	-	0	0	0	2	0.6	2.5			
3	Total	0.6 (mM per 0.3)	100	30	100	0	0	-	20	6	100	80	24	100	6	24	80

Table S8 Overview of biphasic exchange studies and oxidation studies with **TEMPO(BF_4)** of Hydroxymethylfurfural in gel biphasic systems: the gel layer consists of 10%$_{WT}$ PVDF–HFP, 1.2 M LiTFSI in acetonitrile, and the aqueous layer is sat. NaCl, $NaHCO_3$ pH 8.3 in H_2O. In acetonitrile biphasic systems: the organic layer consist of 1.2 M LiTFSI in acetonitrile, and the aqueous layer is sat. $NaHCO_3$ pH 8.3 in H_2O. Hydroxymethylfurfural was added to the aqueous layer *at t*=0 hours. The experiment was done overnight at room temperature. Data is an average of *N*=2. GC analysis was performed after the TMS derivatization procedure on a Trace GC Ultra machine (Interscience) with an RTX-1 column (30 m, 0.25 mm internal diameter, 0.25 µm film thickness), inlet temperature 70°C hold 2.00 minutes ramp 10°C min^{-1} to 340°C.

Entry	Layer measured	Layer size	Hydroxymethylfurfural (HMF) *t=0* hours			2,5-Furandicarboxaldehyde (DFF) *t=0* hours			Hydroxymethylfurfural (HMF) *t=16* hours			2,5-Furandicarboxaldehyde (DFF) *t*=16 hours			Conversion after 16 hours HMF to DFF (µmol)		
		(mL)	mM	µmol	%	mM	µmol	%	mM	µmol	%	mM	µmol	%	HMF	DFF	Conver sion(%)
1	MeCN/ Gel	0.3	0	0	0	0	0	-	92	27.6	96.8	5	1.5	100			
2	Aqueous	0.3	100	30	100	0	0	-	3	0.9	3.2	0	0	0			
3	Total	0.6 (mM per 0.3)	100	30	100	0	0	-	95	28.5	100	5	1.5	100	28.5	1.5	5

Table S9. Dye leaching experiment with ø 0.5 cm FTO|TiO_2|**AP11** in alkaline or organic anolytes. The photoanodes were prepared in the same manner as DSSCs and DSPEC experiments. Plates were soaked overnight in either 1.0 M **TEMPO**, 1.2 M LiTFSI in acetonitrile (3 mL) or 0.1 M glycerol aqueous solution (sat. NaCl, $NaHCO_3$ pH 8.3, 3 mL) to simulate reaction conditions. The experiments were performed in the dark to prevent dye decomposition by over-illumination. After 23 hours, the remaining AP11 on the FTO|TiO_2 electrodes was quantified by dipping the electrodes for 24 hours in a 0.01 M TBAOH in DMF solution (1 mL), after which UV-VIS studies were performed using ε= 19000 cm^{-1} at 364 nm.[1]

Entry	Environment	Dye loading (nmol cm^{-2})
1	Unused	218 (±13.8)
2	Acetonitrile	224 (±15.3)
3	Alkaline aqueous (pH 8.5)	138 (±13.8)

Table S10. Light-driven glycerol oxidation reactions using redox gel DSPEC performed for *t*=23 hours, and some control experiments. The biphasic gel DSPEC: WE electrode compartment consisted of a FTO|TiO_2|**AP11** WE (active area 0.74 cm^2) with a 1.0 M **TEMPO** 3 mm redox-gel layer (10%wt PVDF–HFP, 1.2 M LiTFSI in acetonitrile) and filled with 0.1 M glycerol aqueous solution (sat. NaCl, $NaHCO_3$ pH 8.3, 3 mL). An Ag/AgCl RE which was placed close to the WE. The CE compartment was separated by a Nafion-117 membrane and consisted of an FTO|Pt CE and was filled with 1.0 M AcOH in acetonitrile (3 mL). A bias potential of 0.1 *vs.* Ag/AgCl was applied on the WE (P211 potentiostat, Zahner) and the system was illuminated with a 100 mW cm^{-2} white LED light source (masked size 0.64 cm^2). Differences in control experiments are indicated. Product analysis measured by GC analysis was performed after the TMS derivatization procedure on a Trace GC Ultra machine (Interscience) with an RTX-1 column (30 m, 0.25 mm internal diameter, 0.25 µm film thickness), inlet temperature 70°C hold 2.00 minutes ramp 10°C min^{-1} to 340°C. * Faradaic efficiency was not obtained for a system with photocurrent near 0 µmol.

Entry	Conditions	Glyceraldehyde produced (µmol)	Conversion based on photocurrent (µmol)	Faradaic efficiency (%)	Image of used photoanode
1	Biphasic gel DSPEC	68.3	66.2	~100	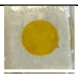
2	w/o light	0	<0	-*	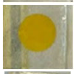
3	w/o **TEMPO**	0	1.00	-*	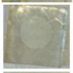
4	w/o gel (aqueous)	7.60	7.40	~100	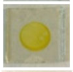
5	Unused	-	-	-	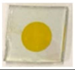

References

[1] R. R. Rodrigues, A. Peddapuram, A. L. Dorris, N. I. Hammer, J. H. Delcamp, *ACS Applied Energy Materials* **2019**, *2*, 5547–5556.
[2] M. A. Mercadante, C. B. Kelly, J. M. Bobbitt, L. J. Tilley, N. E. Leadbeater, *Nature Protocols* **2013**, *8*, 666–676.
[3] M. Suzuka, N. Hayashi, T. Sekiguchi, K. Sumioka, M. Takata, N. Hayo, H. Ikeda, K. Oyaizu, H. Nishide, *Scientific Reports* **2016**, *6*, 28022–28022.
[4] P. Podolec, A. H. Szabó, J. Blaško, R. Kubinec, R. Górová, J. Višňovský, A. Gnipová, A. Horváth, V. Bierhanzl, T. Hložek, R. Čabala, *Journal of Chromatography B: Analytical Technologies in the Biomedical and Life Sciences* **2014**, *967*, 134–138.
[5] E. Rojas-Escudero, A. L. Alarcón-Jiménez, P. Elizalde-Galván, F. Rojo-Callejas, in *Journal of Chromatography A*, Elsevier, **2004**, pp. 117–120.
[6] D. Gardiner, *Carbohydrate Research* **1966**, *2*, 234–239.
[7] V. A. Yaylayan, S. Harty-Majors, A. A. Ismail, *Carbohydrate Research* **1999**, *318*, 20–25.
[8] A. Parodi, E. Diguilio, S. Renzini, I. Magario, *Carbohydrate Research* **2020**, *487*, 107885.
[9] H. Cheema, K. S. Joya, in *Titanium Dioxide - Material for a Sustainable Environment*, InTech, **2018**.
[10] D. F. Bruggeman, T. M. A. Bakker, S. Mathew, J. N. H. Reek, *Chemistry - A European Journal* **2021**, *27*, 218–221.
[11] D. F. Bruggeman, S. Mathew, R. J. Detz, J. N. H. Reek, *Sustainable Energy and Fuels* **2021**, *5*, 5707–5716.
[12] R. Ciriminna, M. Pagliaro, *Advanced Synthesis and Catalysis* **2003**, *345*, 383–388.
[13] M. A. Bajada, S. Roy, J. Warnan, K. Abdiaziz, A. Wagner, M. M. Roessler, E. Reisner, *Angewandte Chemie - International Edition* **2020**, *59*, 15633–15641.

Author Contributions

D. F. Bruggeman designed & performed experiments, interpreted data, and wrote the manuscript. A. A. H. Laporte performed experiments, interpreted data, and contributed to the manuscript. S. Mathew provided expertise in the design and experimental work and contributed to the manuscript. R. J. Detz conceived the project and contributed to the manuscript, and J. N. H. Reek conceived the project, supervised, and contributed to the manuscript.
